# Supplementary material for: Parthenolide disrupts mitosis by inhibiting ZNF207/BUGZ-promoted kinetochore-microtubule attachment
Source: EMBO J. 2025 May 27;44(13):3764–93. doi: 10.1038/s44318-025-00469-2 (PMC12219771; doi:10.1038/s44318-025-00469-2)
Supplement: Supplementary file 1 — Appendix [file 44318_2025_469_MOESM1_ESM.pdf]

Appendix for

"Parthenolide disrupts mitosis by inhibiting ZNF207/BUGZ-promoted kinetochore-microtubule attachment"

Table of content:

Appendix Figure S1 2
Appendix Figure S2 4
Appendix Figure S3 6
Appendix Figure S4 8
Appendix Figure S5 10
Appendix Figure S6 12
Appendix Figure S7 14
Appendix Supplementary Methods 16

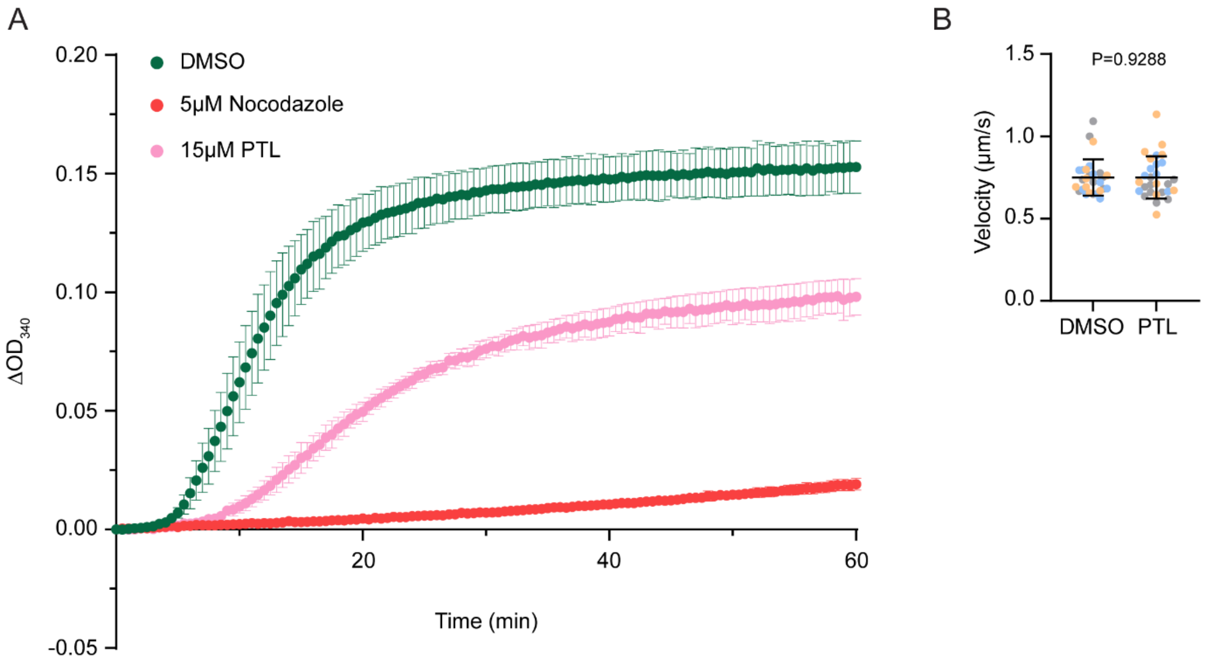

Appendix Figure S1

Appendix Figure S1. A) In vitro microtubule polymerization assay with indicated compounds. B) Quantification of the velocity of EB1-GFP comets from U2OS EB1-GFP cells undergoing the indicated treatments. N, n (N = number of cells, n = number of experiments) DMSO (28, 3), 15 µM PTL (28, 3). Replicates in B are color coded. All data are presented as mean and SD values from three independent replicates. Statistical analysis was performed by using non-parametric Mann-Whitney test.

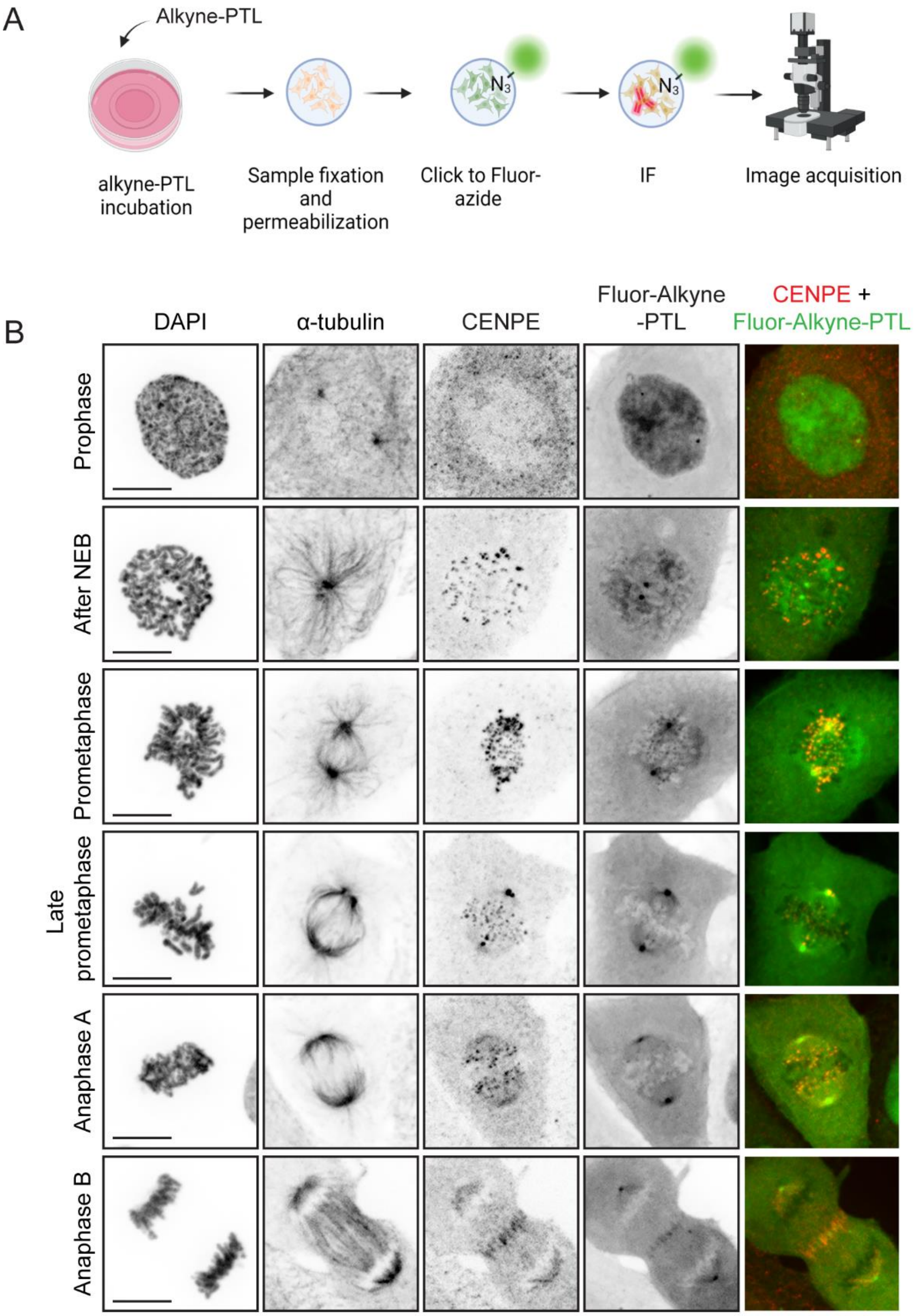

Appendix Figure S2

Appendix Figure S2. A) Illustrative scheme of the procedure for click-based imaging of alkyne-PTL. Briefly, cells were treated with alkyne-PTL prior PFA fixation. After fixation, click reaction with fluor-488 or fluor-Cy5 azide was performed over the coverslips for 1 h at RT, followed by immunofluoresce against the desired proteins and confocal imaging. B) Spinning-disk confocal images of different mitotic stages in U2OS cells stained for 5 µM fluor-488-alkyne-PTL, tubulin and CENP-E. Scale bar: 10 µm.

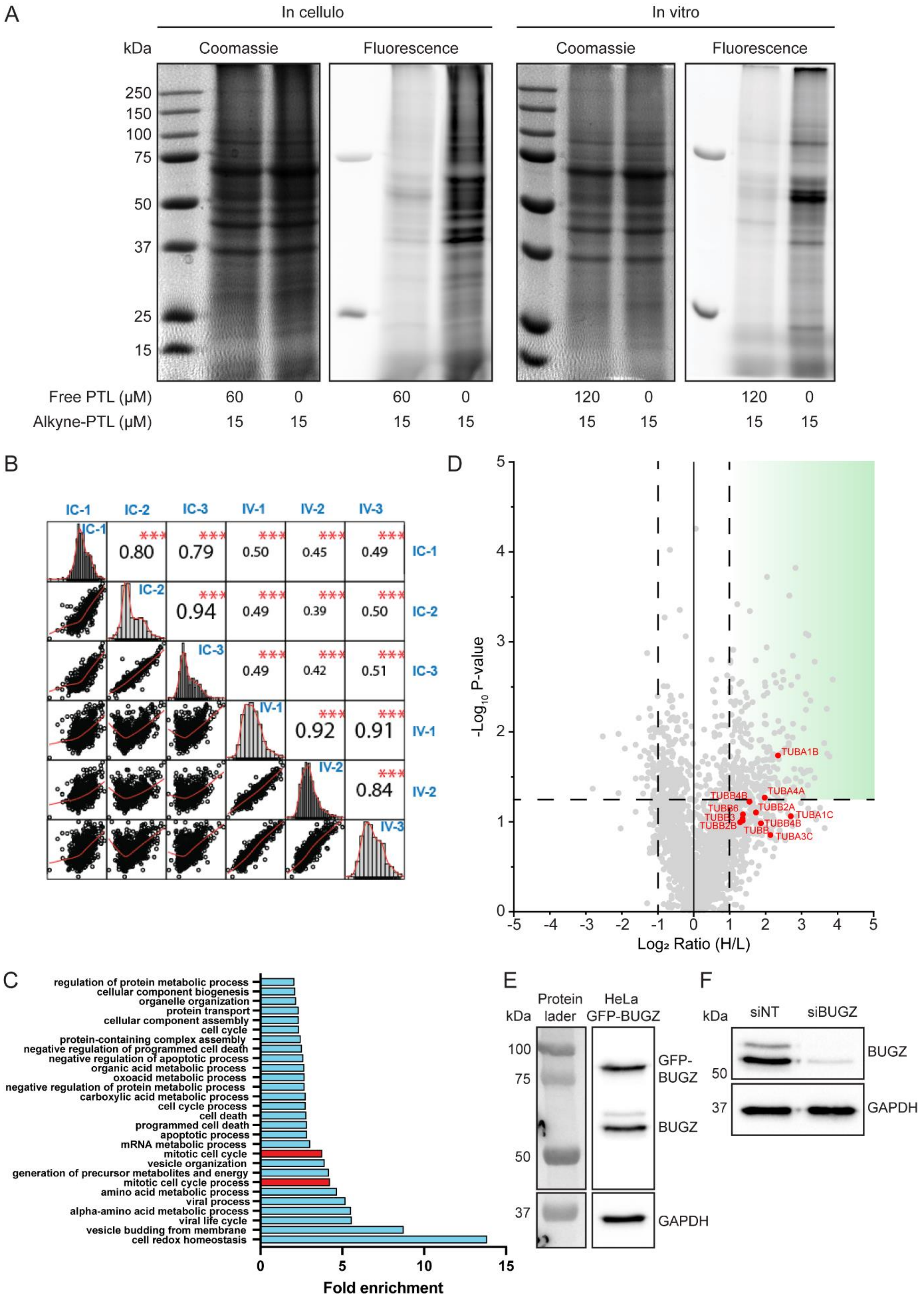

Appendix Figure S3

Appendix Figure S3. A) Fluorencence and Coomassie staining of U2OS cell lysate containing alkyne-PTL. B) Comparative table showing the correlation between experiments and replicates. IC= *in cellulo*. IV= *in vitro*. C) GO-enrichment analysis of the 300 filtered proteins according to their cellular localization. D) Volcano plot showing quantitative mass-spectrometry results for *in vitro* procedure. Dashed horizontal line shows the p-value cut-off ($p<0.05$) and vertical dashed lines indicate the upregulated/downregulated (competed/ non competed by free PTL) proteins. The green transparent region groups all the proteins that satisfy the p-value cut-off and are upregulated (competed) with a SILAC ratio higher than 2. N (number of experiments): 3. Statistical analysis was performed using unpaired t-test. E) Western-blot with anti-BUGZ antibody of protein extract from HeLa cells stably expressing GFP-BUGZ. F) Immunoblot for BUGZ depletion efficiency.

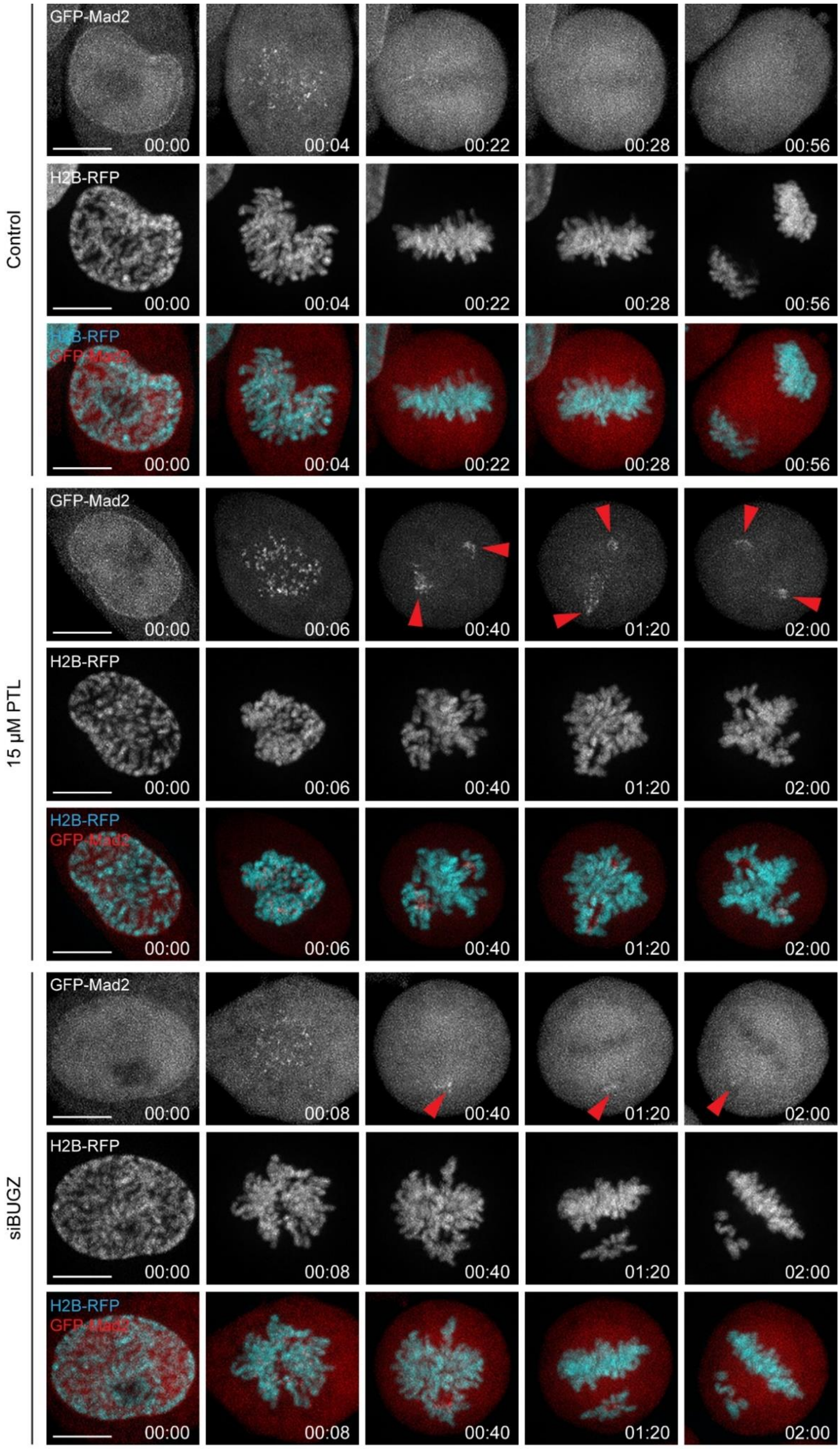

Appendix Figure S4

Appendix Figure S4. Representative spinning disk confocal time-series of mitosis in HeLa cells stably expressing GFP-MAD2 and infected with adenovirus to express H2B-RFP following indicated treatments. GFP-MAD2 signal at the kinetochores of uncongressed chromosomes is highlighted with red arrowheads. Scale bar: 10 µm.

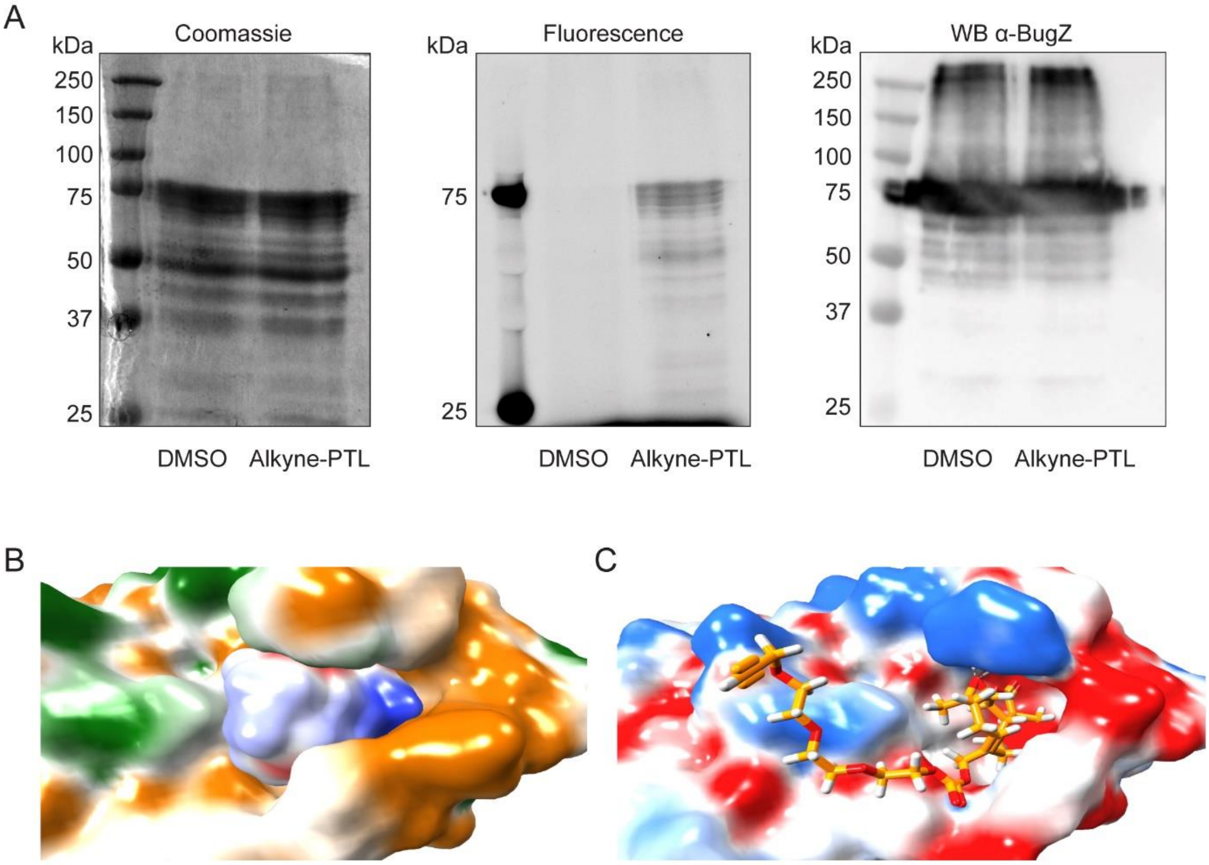

Appendix Figure S5

Appendix Figure S5. A) From left to right, Coomassie staining, fluorescence and immunoblot of purified GST-BUGZ incubated with DMSO or alkyne-PTL. B) AlphaFold 3-based surface representation of Cys54 bound PTL displaying electrostatic properties. Red and orange illustrate negative and blue and green positive potentials. C) AlphaFold 3 model representing binding pocket with conjugated and Cys54 bound parthenolide in the binding pocket showing ample space to accommodate the alkyne conjugate.

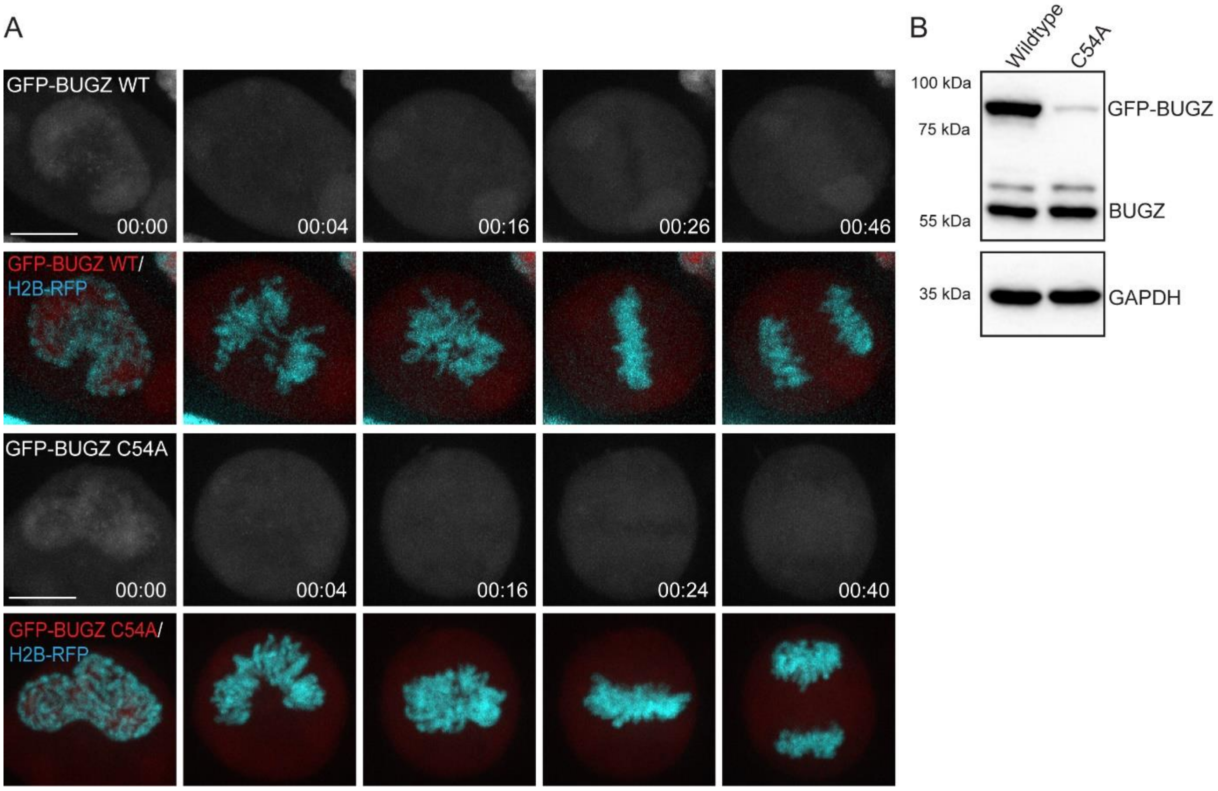

Appendix Figure S6

Appendix Figure S6. A) Representative spinning disk confocal time-series of mitosis in HeLa cells stably expressing GFP-BUGZ wildtype (WT) or C54A mutant and infected with adenovirus for expression of H2B-RFP. Scale bar: 10 µm. B) Immunoblot with anti-BUGZ antibody of protein extract from HeLa cells stably expressing GFP-BUGZ WT or mutant.

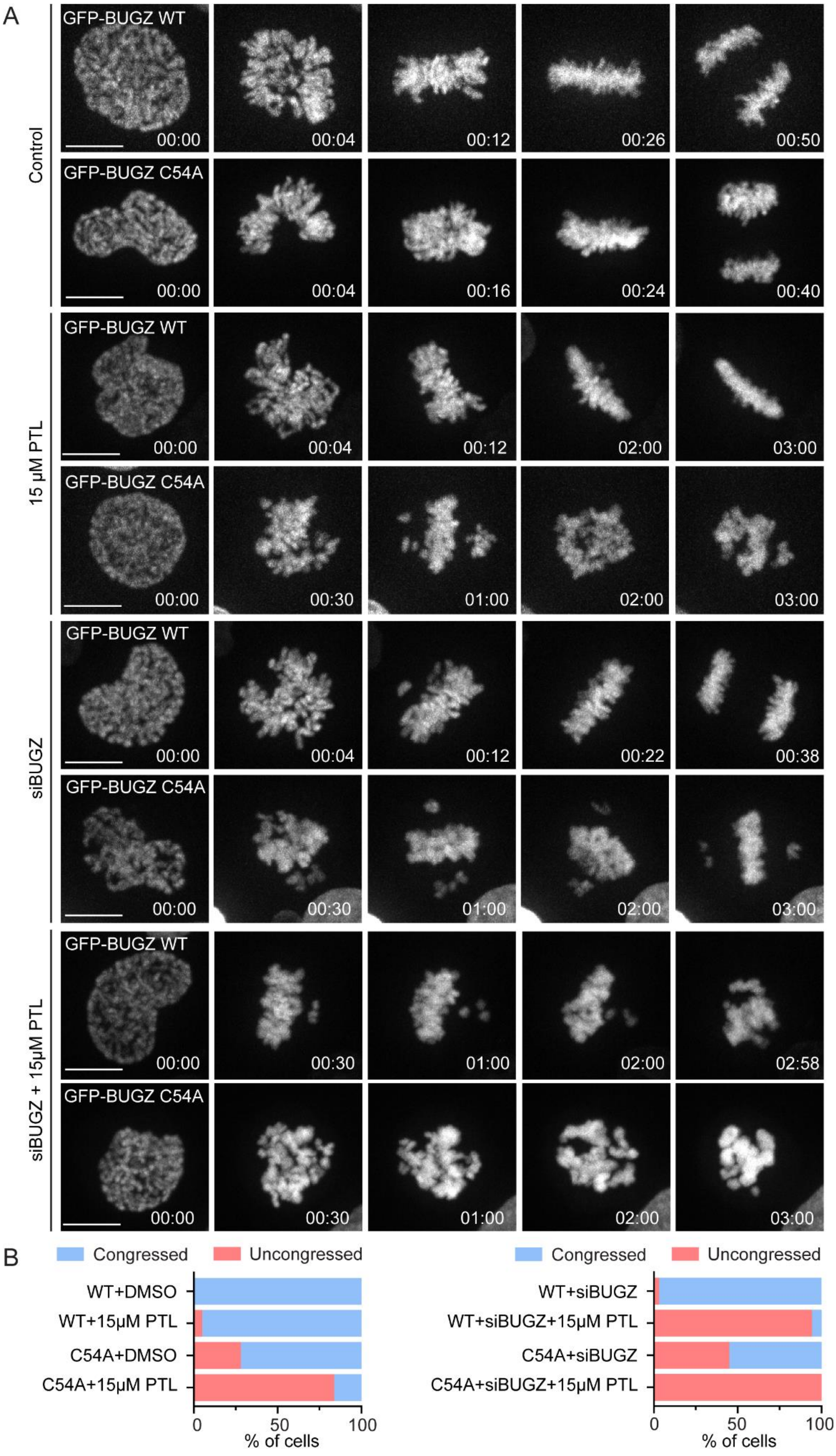

Appendix Figure S7

Appendix Figure S7. A) Representative spinning disk confocal time-series of mitosis in HeLa cells stably expressing RNAi-resistant GFP-BUGZ wildtype or C54A mutant, infected with adenovirus for expression of H2B-RFP and undergoing the indicated conditions. Scale bar: 10 µm. B) Quantification of chromosome congression status in cells with the conditions indicated in A. N, n (N = number of cells, n = number of experiments): GFP-BUGZ WT + DMSO (57, 3), GFP-BUGZ WT + 15 µM PTL (97, 3), GFP-BUGZ C54A + DMSO (61, 3), GFP-BUGZ C54A + 15 µM PTL (46, 3), GFP-BUGZ WT + siBUGZ (102, 3), GFP-BUGZ WT + siBUGZ + 15 µM PTL (68, 3), GFP-BUGZ C54A + siBUGZ (34, 3), GFP-BUGZ C54A + siBUGZ + 15 µM PTL (28, 3).

Appendix Supplementary Methods

Synthesis of Alkyne terminated – PEG Carbonate ester analog of Parthenolide.

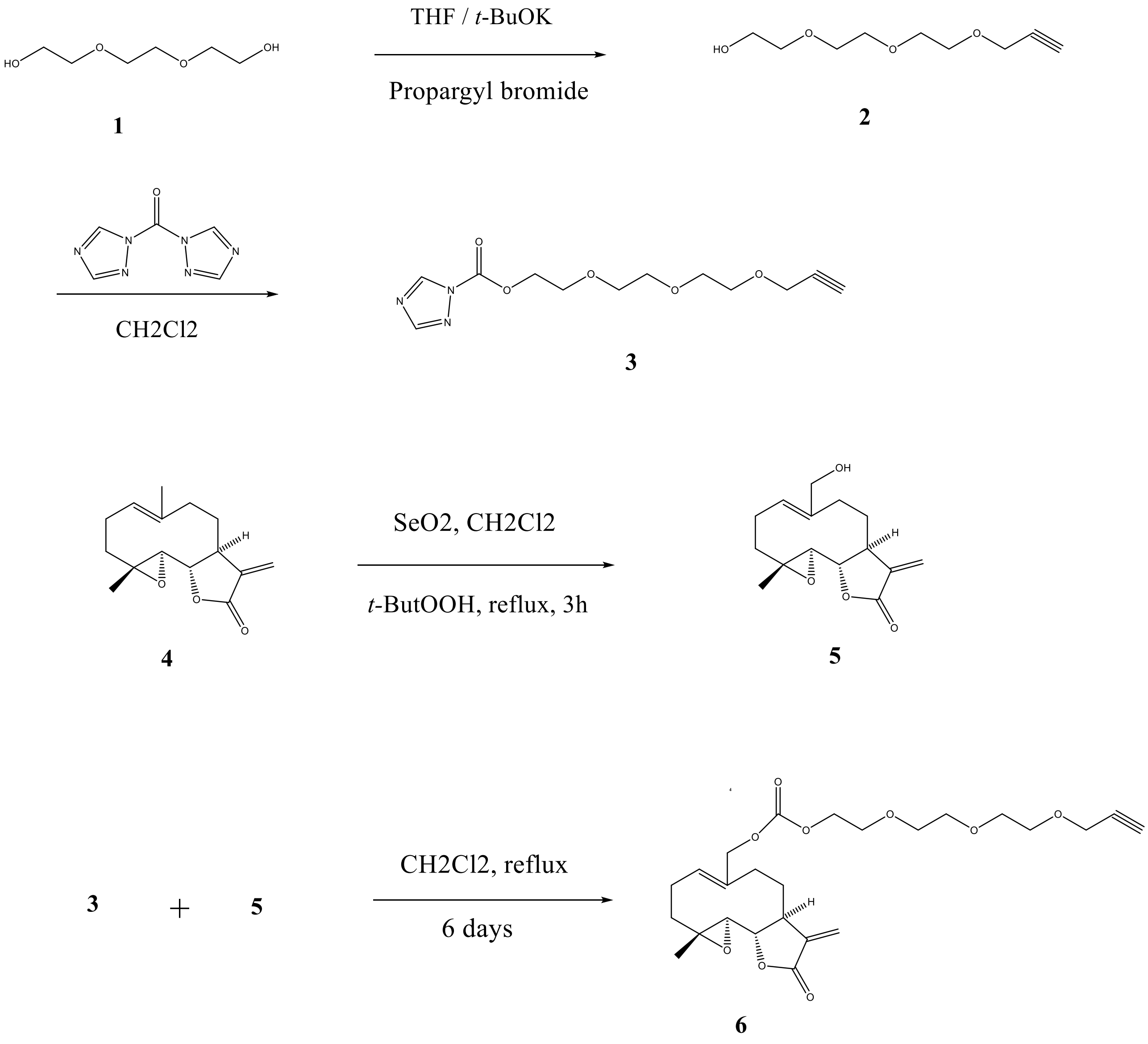

Synthesis of Alkyne terminated – PEG Carbonate analog of Parthenolide scheme.

Experimental procedures:

Parthenolide was purchased from AEchem Scientific Corporation USA, 1,1´-carbonylbis(1,2,4-troazole) from Fluorochem UK. All other reagents and solvents were analytical grades purchased from Sigma-Aldrich Chemical Co. and used as received unless otherwise stated. Merck silica gel 60 (0.040-0.060 mm) was used for flash chromatography purification of the products. ^{1}H NMR and ^{13}C NMR spectra were recorded at 500 MHz and 126 MHz on a Brucker Ultrashield Plus 500 spectrometer using $CDCl_3$ as a solvent and TMS as internal standard. HRMS LC-MS was carried out on a Brucker MicrOTOF-Qll-system with ESI-source with nebulizer 1.2 bar, dry gas 8.0 l/min, dry temperature 200 ^{o}C, capillary 4500 V, end plate offset -500 V.

2-(2-(2-(Prop-2-yn-1-yloxy)ethoxy)ethoxy)ethanol **2**[1]:

Into a suspension of *t*-BuOK (3.82 g, 34.0 mmol) in anhydrous THF (150 ml) was added a solution of tri(ethylene)glycol **1** (9.82 g, 65.4 mmol) dissolved in anhydrous THF (30 ml) at 0 ^{o}C. The mixture was allowed to stir at room temperature for 30 minutes then propargyl bromide (3.89 g, 32.7 mmol) was added dropwise via cannula under nitrogen atmosphere. The resulting mixture was allowed to stir at room temperature for 20 hours. After completion of the reaction, the mixture was diluted with THF (150 ml) and filtered through a plug of Celite. The filtrate was concentrated and the residue was purified by column chromatography on silica gel, using EtOAc as eluent. The product was a viscous pale yellow liquid (4.31 g, 85%). ^{1}H NMR (500 MHz, $CDCl_3$) δ 4.14 (d, 2H), 3.66-3.63 (m, 10H), 3.55 (m, 2H), 2.93 (bs, 1H), 2.41 (t, 1H). ^{13}C NMR (126 MHz $CDCl_3$) δ 74.9, 72.5, 72.4, 70.6, 70.3, 70.2, 69.0, 61.7, 58.4. LC-MS (ESI), *m/z* 189.10 $(M+H)^+$.

2-(2-(2-(Prop-2-yn-1-yloxy)ethoxy)ethoxy)ethyl-1,2,4-triazole-1-carboxylate **3**:

To a stirred solution of alkyne-PEG **2** (0.460 g, 2.45 mmol) in dichloromethane (20 ml) was added bis-1,1´-carbonyltriazole (0.685 g, 3.68 mmol) under nitrogen atmosphere at room temperature, and the resulting reaction mixture was stirred for 12 hours. After completion of the reaction, water (40 ml) was added and the mixture was extracted with dichloromethane (2×50 ml). The organic phase was washed with water (2×20 ml), dried over anhydrous sodium sulfate and concentrated under reduced pressure to afford a pale yellow viscous liquid. This was purified by column chromatography on silica gel using EtOAc as eluent to afford pure **3** as colorless viscous liquid (0.126 g, 18%). This material was used without further purification in the next step. ^{1}H NMR (500 MHz, $CDCl_3$) δ 8.82 (s, 1H), 8.04 (s, 1H), 4.62 (m, 2H), 4.16 (t, 2H), 3.84 (m, 10H), 2.40 (t, 1H). LC-MS (ESI), *m/z* 284.10 $(M+H)^+$.

Melampomagnolide **5**[2]**:**

A solution of Parthenolide **4** (0.622 g, 2.5 mmol) in dichloromethane (25 ml) was treated with SeO_2 (0.340 g, 2.5 mmol) and *t*-butyl hydroperoxide (0.15 ml, 5-6 M in decane) and the mixture was refluxed gently for 1.5 hours, after which was cooled and decanted and the solid residue was rinsed with dichloromethane (2×20 ml). the combined organics was concentrated under reduced pressure and the solid residue was subjected to column chromatography on silica gel using EtOAc-heptane (2:1 to 3:1) as eluent to afford pure **5** as a beige solid (0.42 mg, 62%). ^{1}H NMR (500 MHz, $CDCl_3$) δ 6.23 (d, 1H), 5.65 (t, 1H), 5.55 (d, 1H), 4.13 (q, 2H), 3.85 (t, 1H), 2.86 (m, 2H), 2.41 (m, 5H), 2.16 (m, 2H), 1.54 (s,3H). ^{13}C NMR (126 MHz $CDCl_3$) δ 169.5, 139.6, 138.9, 127.4, 120.2, 81.2, 65.8, 63.4, 60.1, 42.8, 36.8, 25.6, 24.0, 23.8, 18.0. LC-MS (ESI), *m/z* 265.10 $(M+H)^+$.

Alkyne – terminated PEG carbonate ester of Melampomagnolide **6**

To the triazole alkyne-PEG carbonate **3** (0.0506 g, 0.18 mmol) in dichloromethane (2.5 ml) was added Melampomagnolide **5** (0.0423 g, 0.16 mmol) in dichloromethane (2.5 ml) at room temperature under nitrogen atmosphere. The reaction mixture was heated to reflux for 6 days, monitoring the reaction mixture by HPLC analysis. The mixture was cooled to room temperature and the crude product was subjected to column chromatography on silica gel using EtOAc-dichloromethane (3:1) to afford an eluting fraction, which afforded compound **6** (0.0562 g, 73%) as a colorless viscous liquid. ^{1}H NMR (500 MHz, $CDCl_3$) δ 6.21 (d, 1H), 5.71 (t, 1H), 5.53 (d, 1H), 4.64 (m, 1H), 4.55 (m, 1H), 4.24 (m, 2H), 4.16 (m, 2H), 3.82 (t, 1H), 3.65-3.7 (m, 9H), 2.82 (m, 2H), 2.41-2.11 (m, 8H), 1.67 (m, 1H), 1.52 (s, 3H), 1.08 (t, 1H). ^{13}C NMR (126 MHz $CDCl_3$) δ 169.4, 155.0, 138.7, 134.5, 131.5, 120.4, 81.0, 79.7 (2 signals), 74.6, 70.7, 70.5, 70.4, 69.1, 68.9, 67.3, 63.3, 60.0, 58.4, 42.7, 36.6, 25.8, 24.3, 23.7, 18.0. LC-MS (ESI), *m/z* 479.21 $(M+H)^+$.

References:

1. V. Percec *et al.*, *J. Am. Chem. Soc*. **2013**, *135 (24)*, 9055-9077.
2. S. Nasim, S.n Pei, F. K. Hagen, G. T. Jordan, P. A. Crooks. *Bioorg. Med. Chem*. **2011**, ffff*19*, 1515-1519.

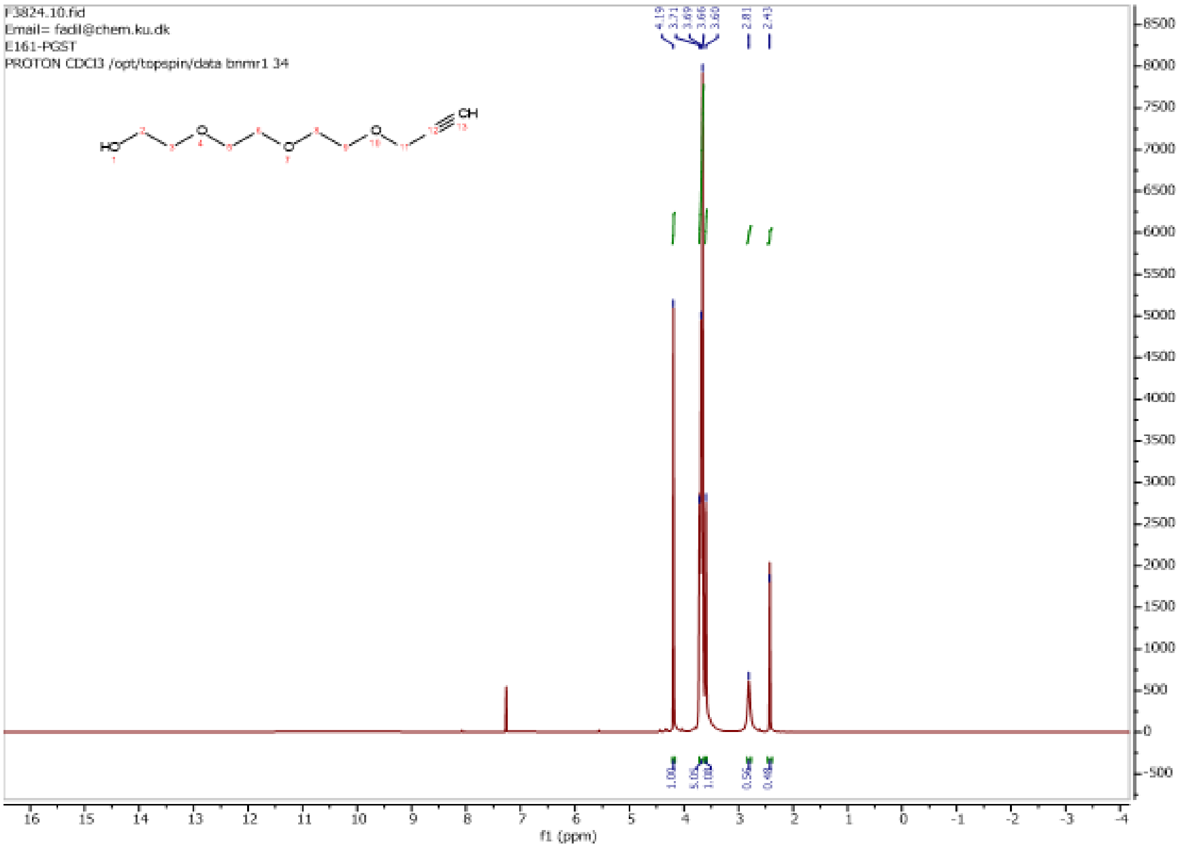

^{1}H NMR (500 MHz, CDCl3) for compound 2.

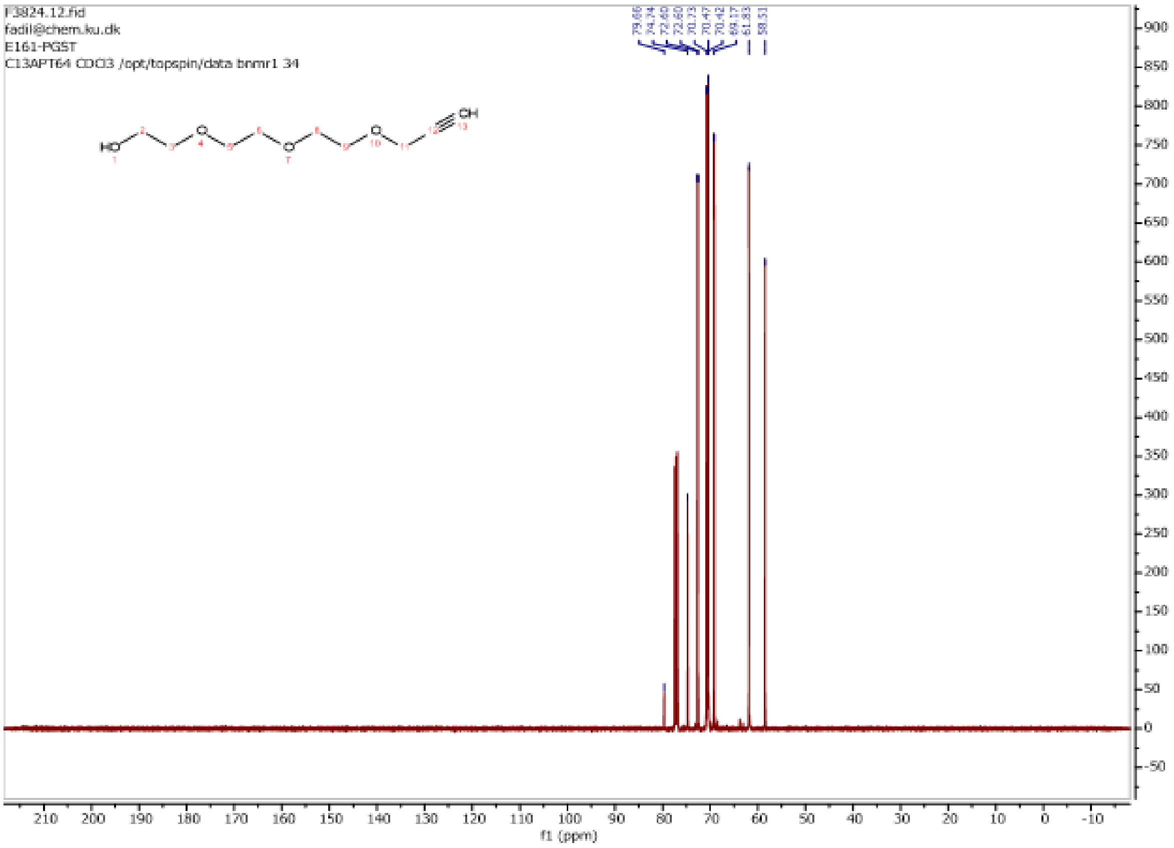

^{13}C NMR (126 MHz, CDCl3) for compound 2.

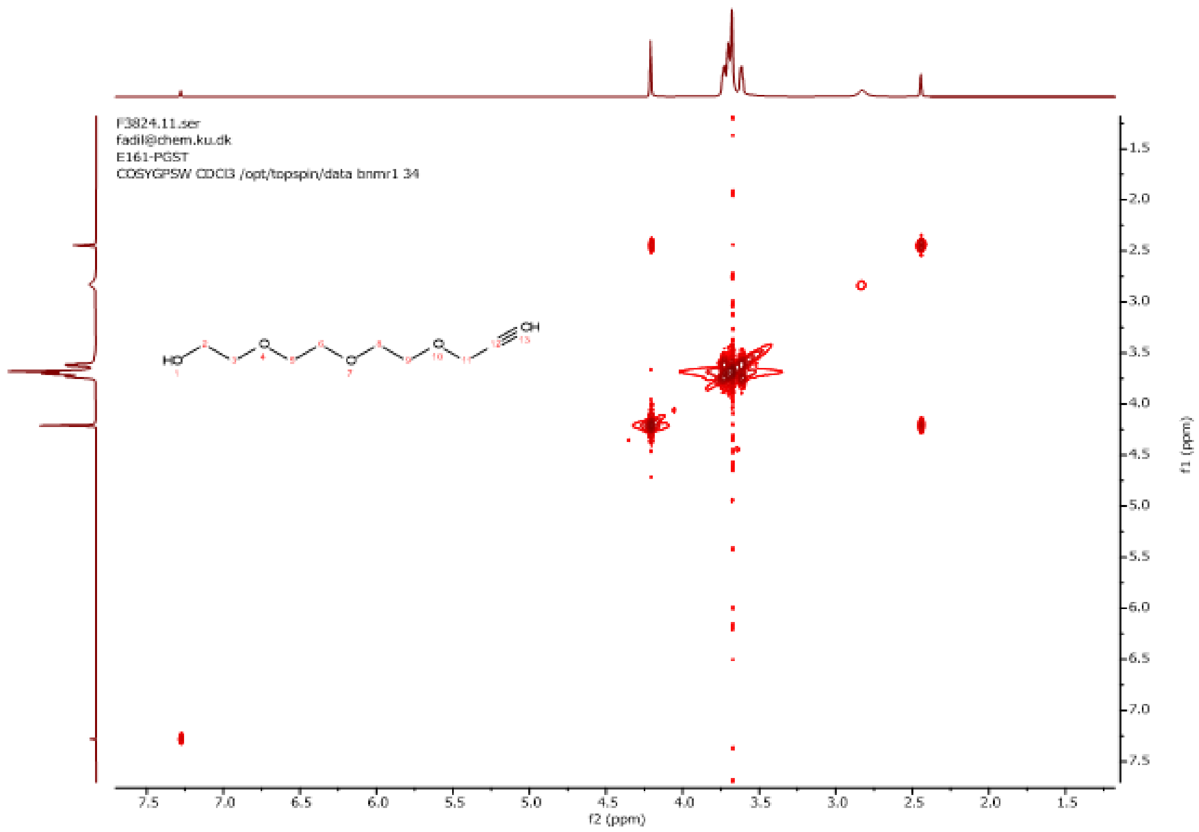

Cosy spectra for compound 2.

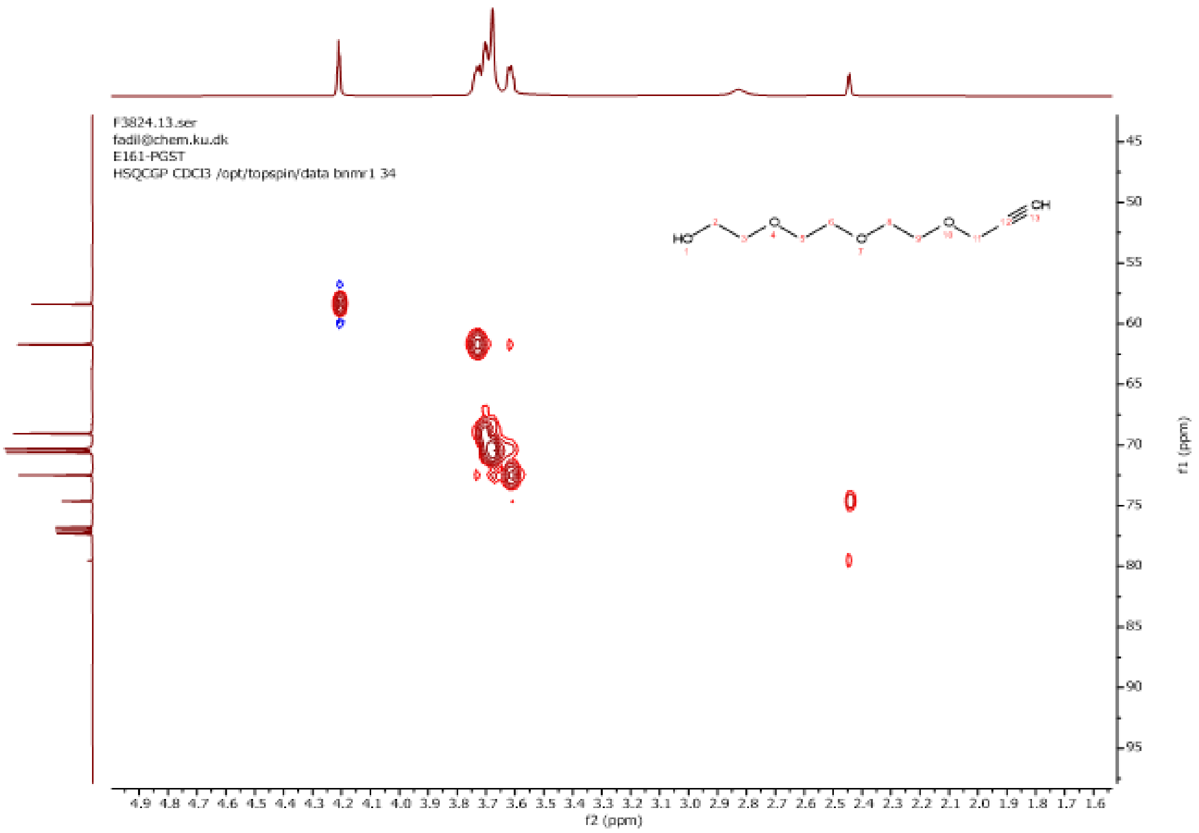

HSQC spectra for compound 2.

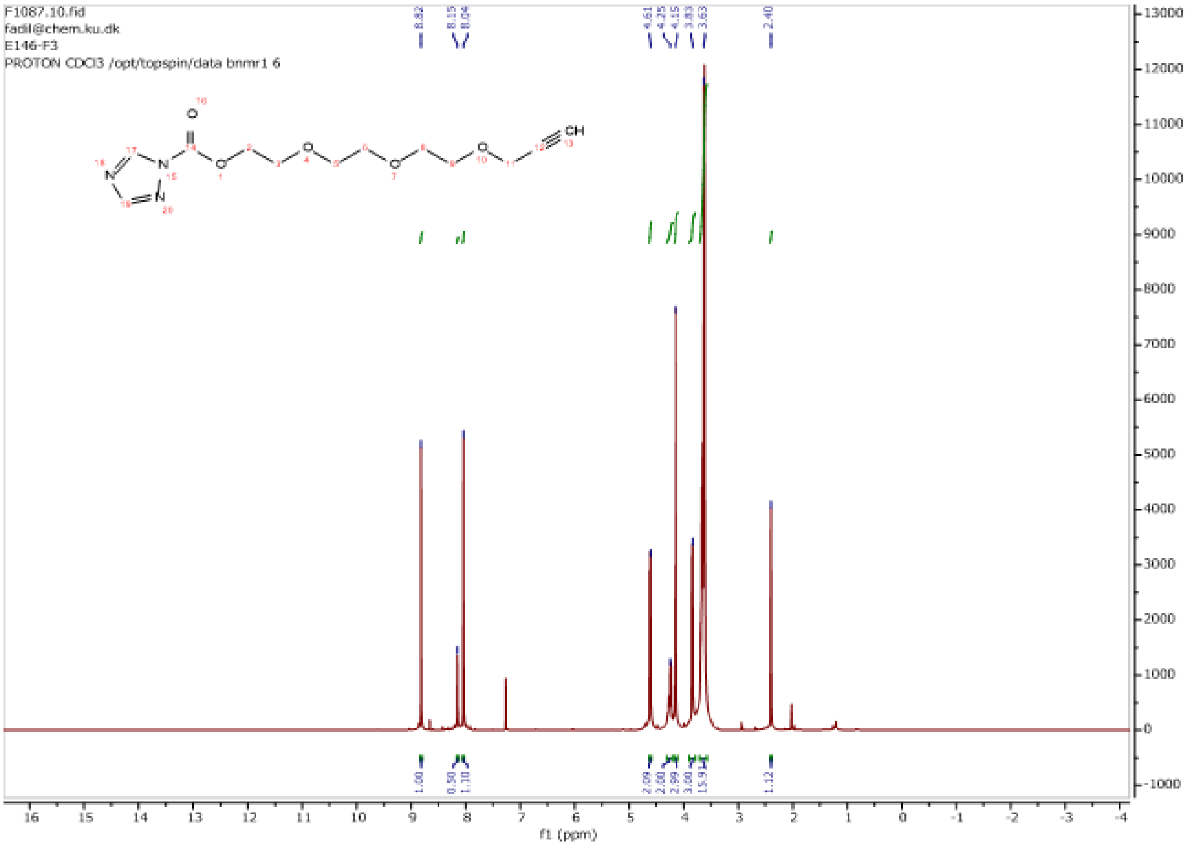

^{1}H NMR (500 MHz, CDCl3) for compound 3.

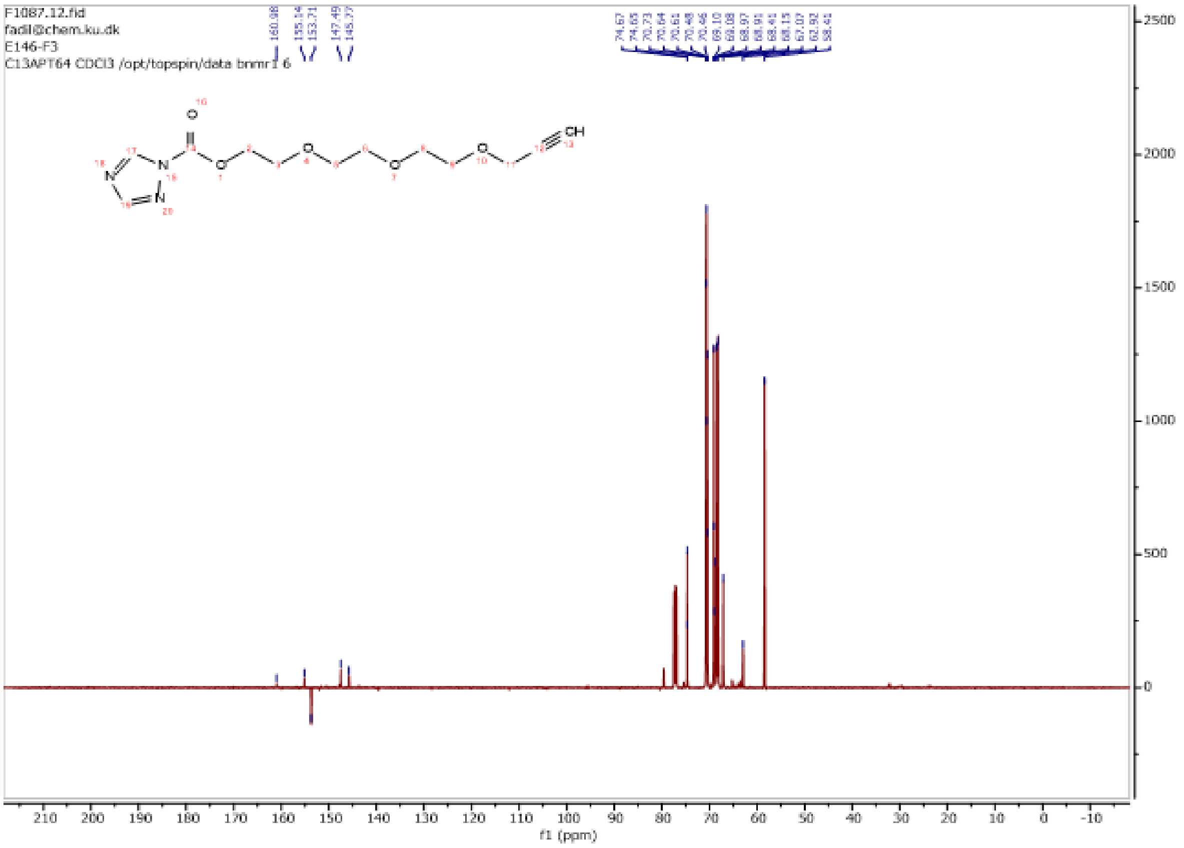

^{13}C NMR (126 MHz, CDCl3) for compound 3.

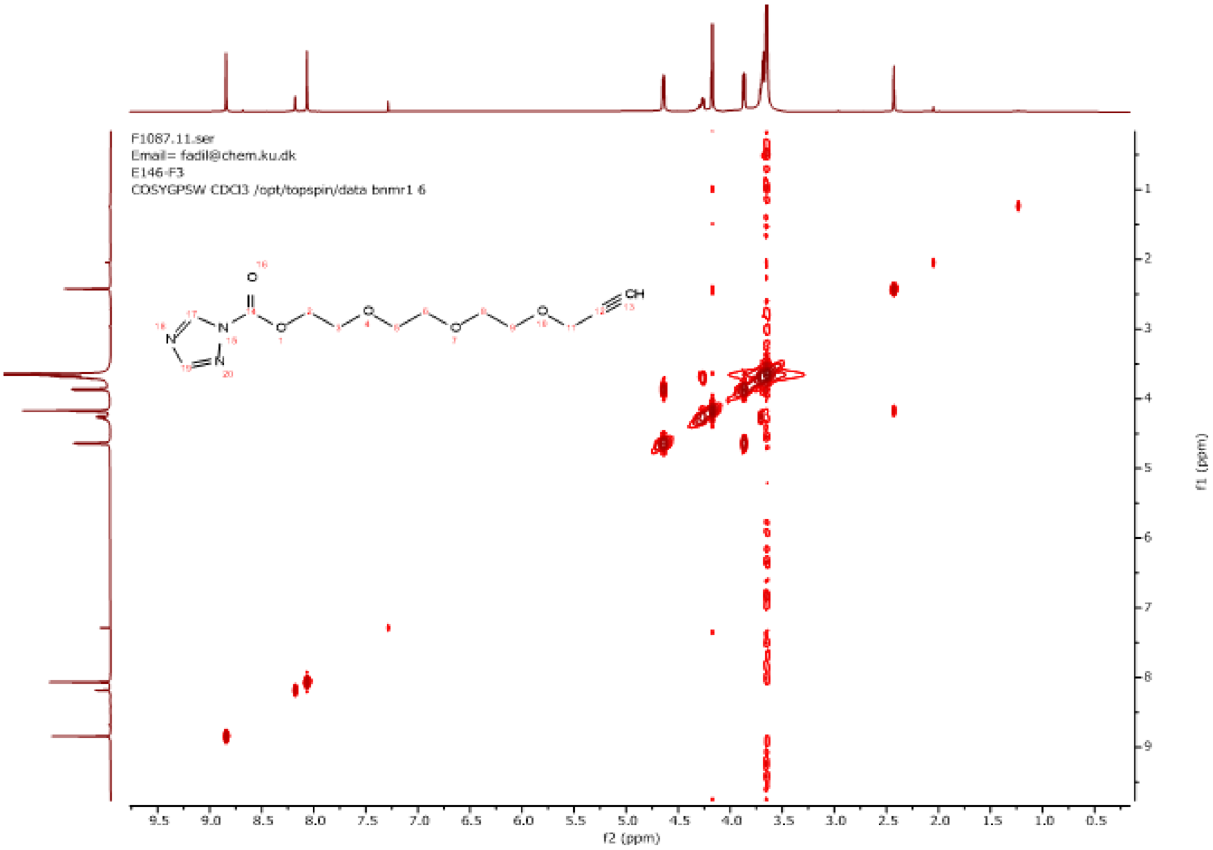

Cosy spectra for compound 3.

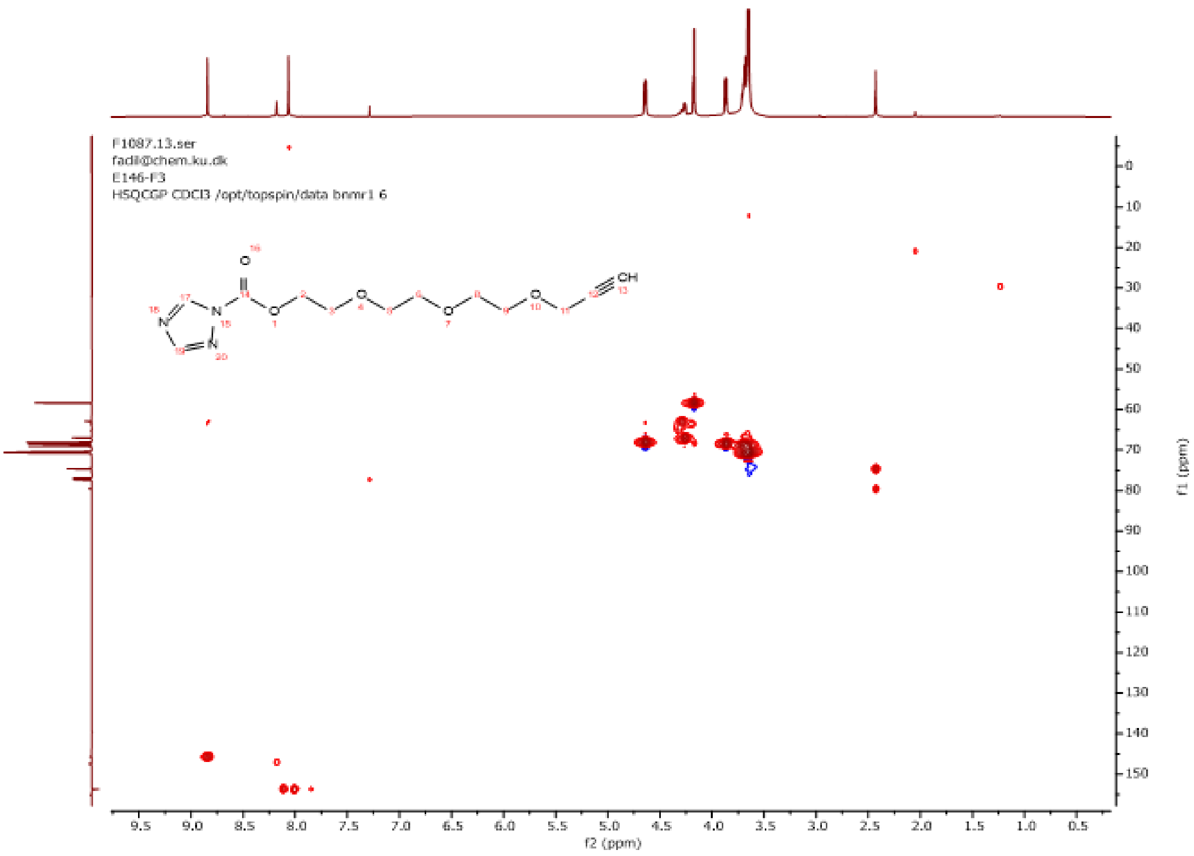

HSQC spectra for compound 3.

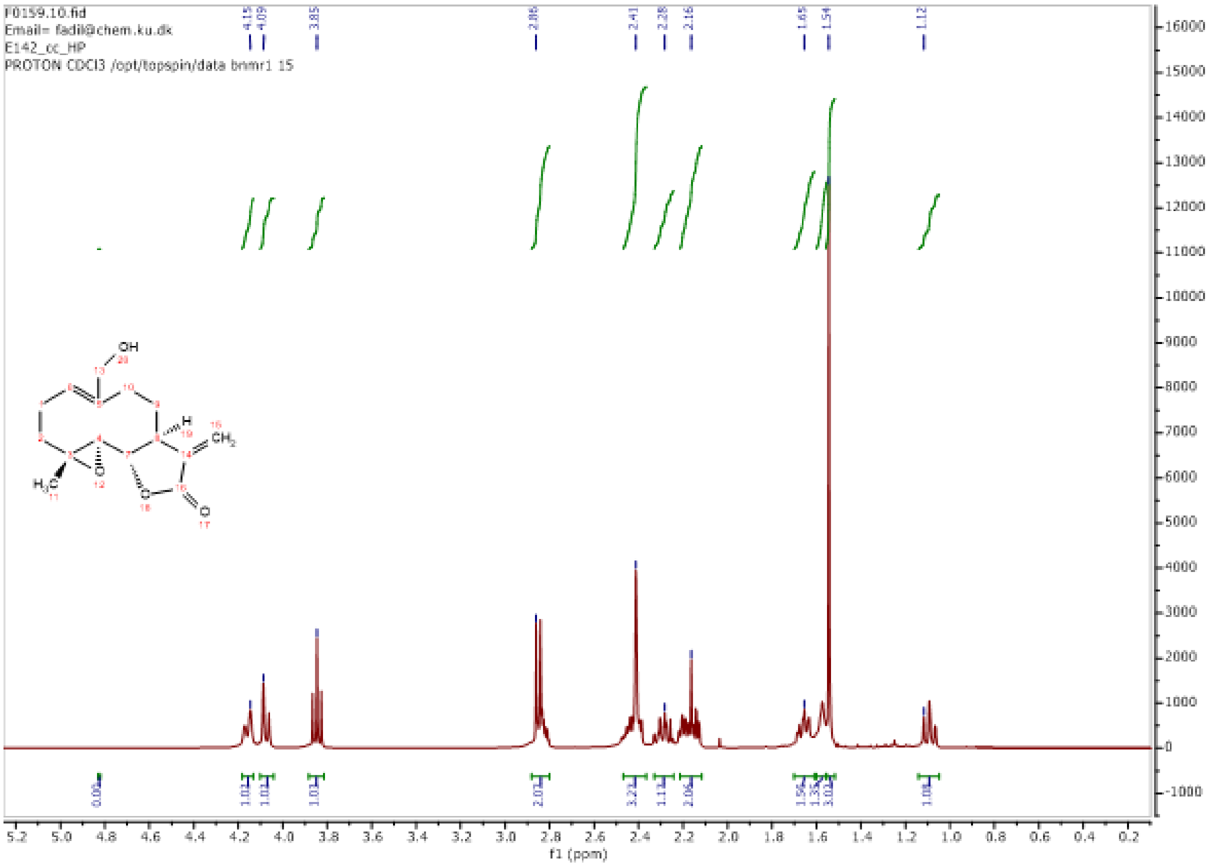

^{1}H NMR (500 MHz, CDCl3) for compound 5.

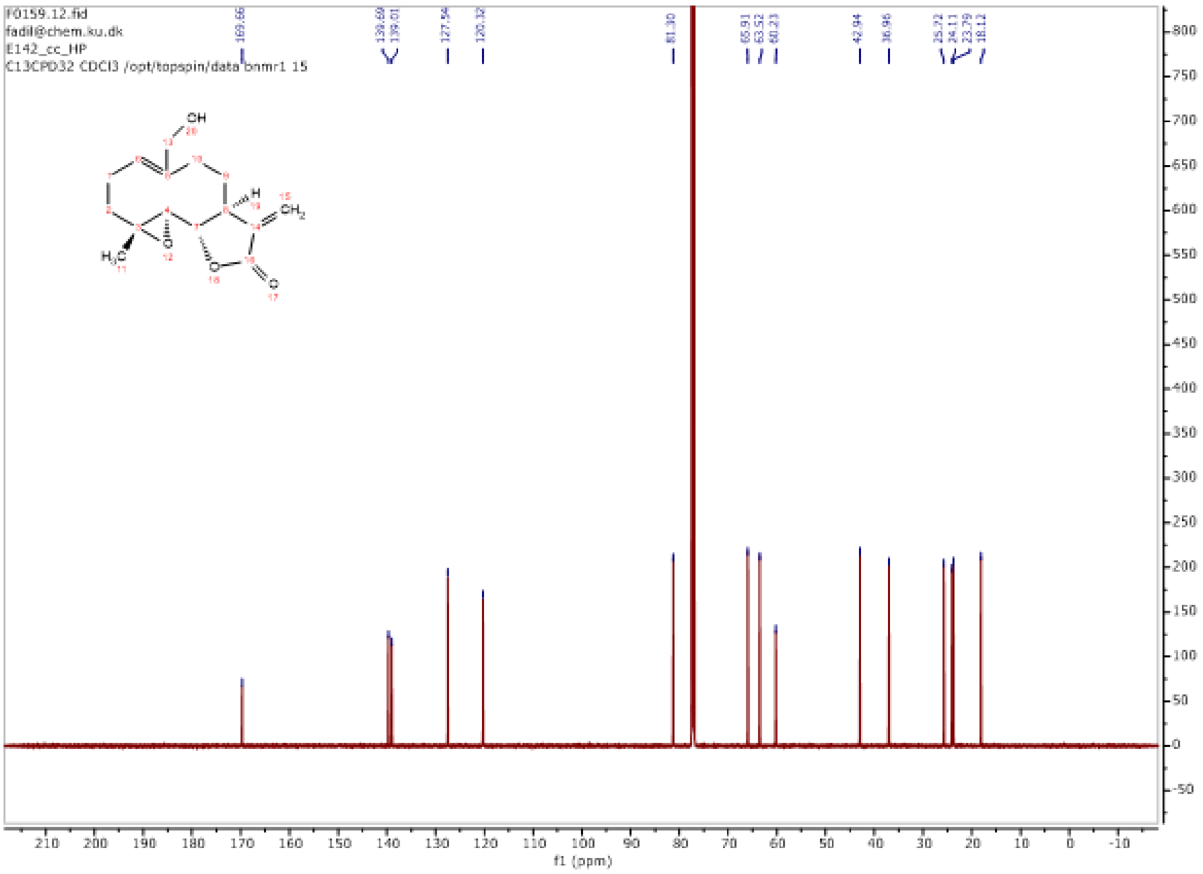

^{13}C NMR (126 MHz, CDCl3) for compound 5.

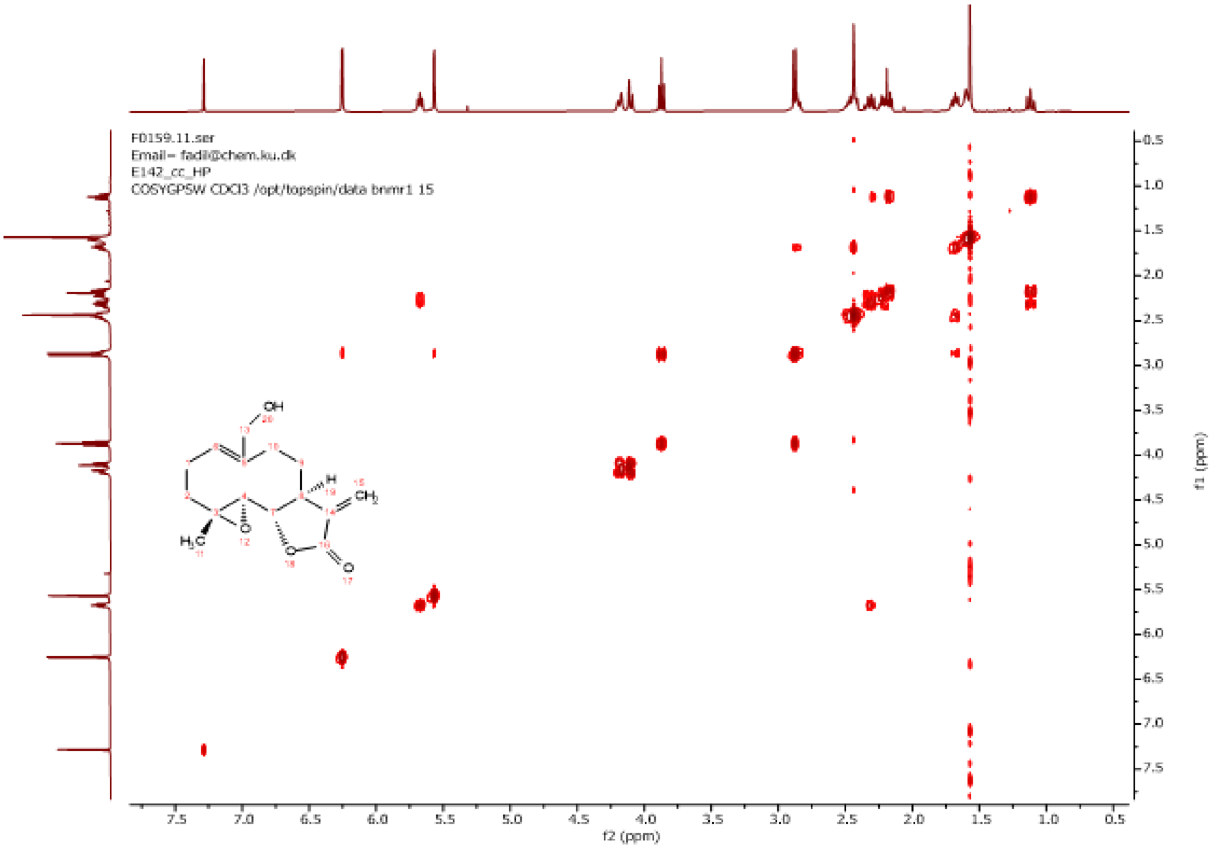

Cosy spectra for compound 5.

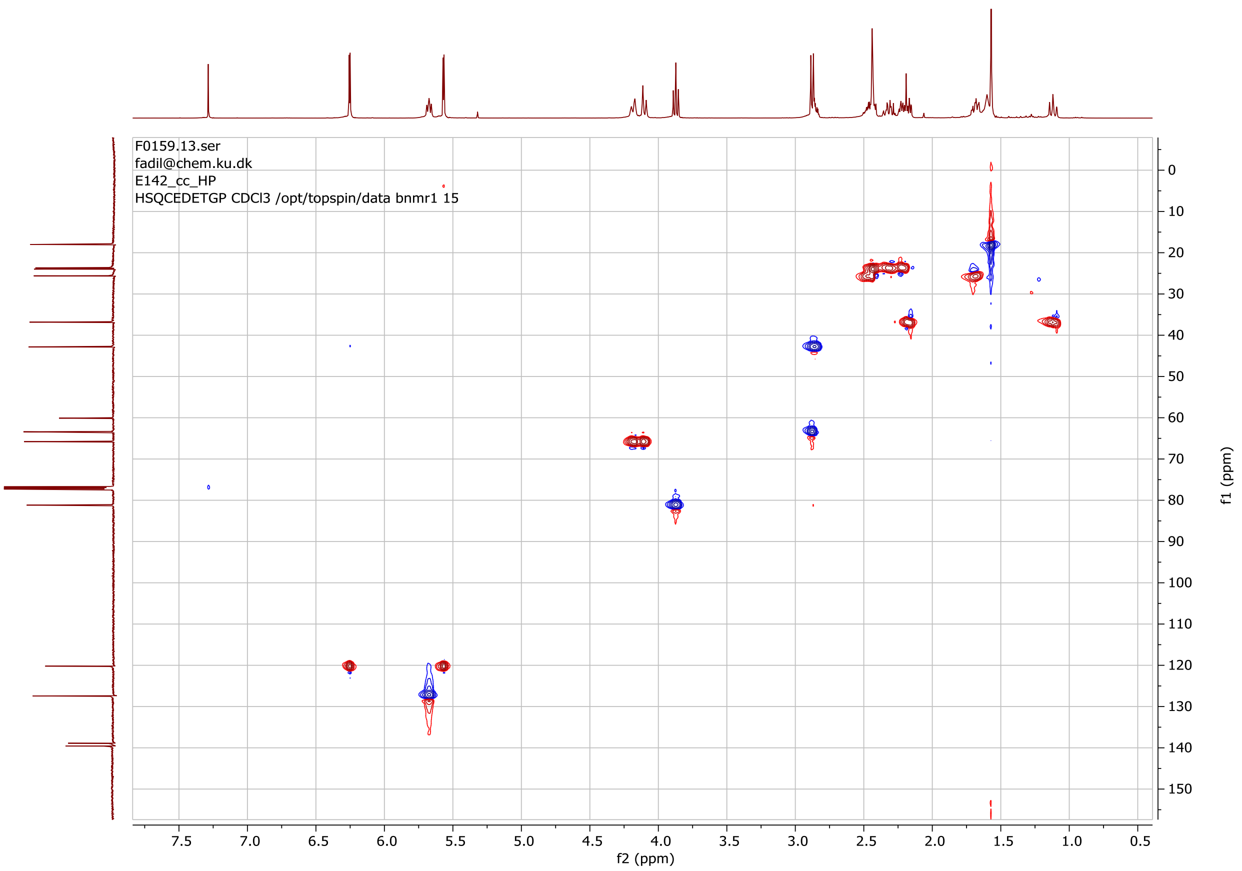

HSQC spectra for compound 5.

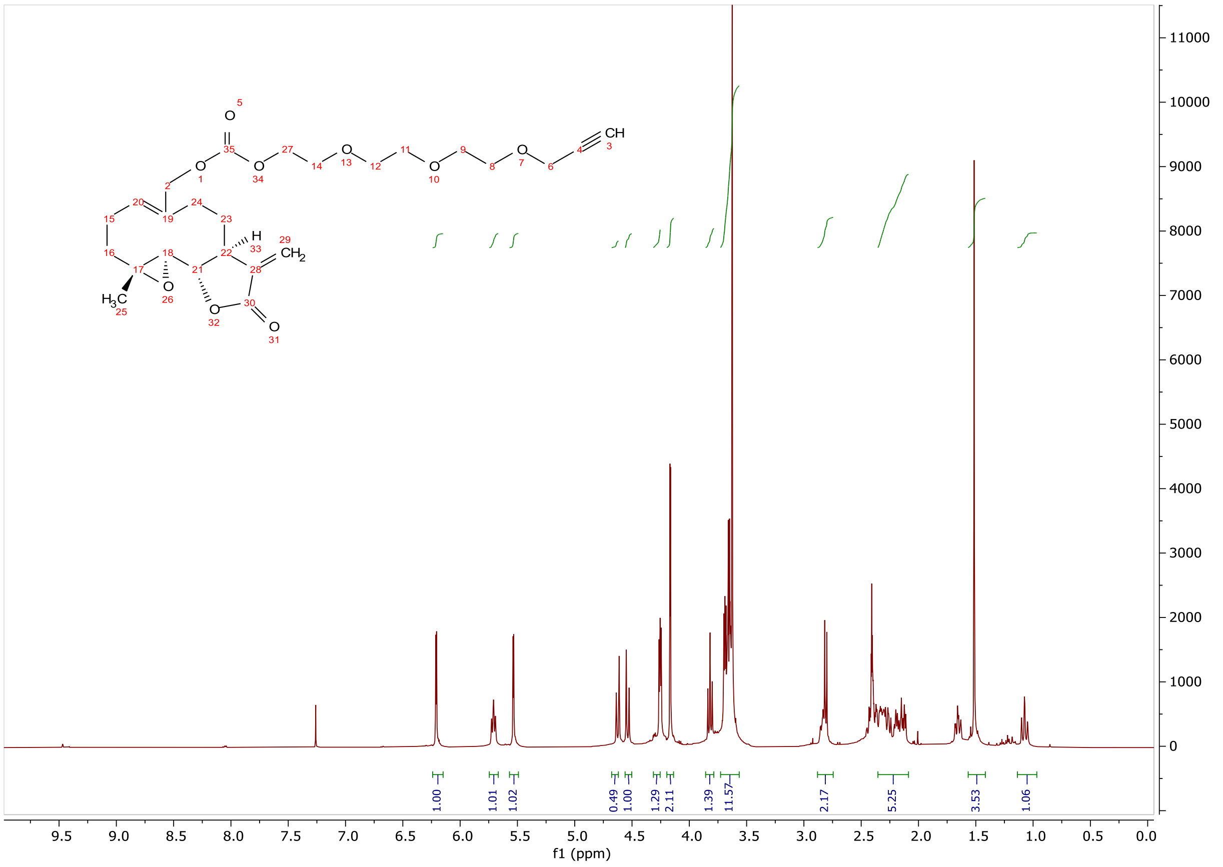

^{1}H NMR (500 MHz, CDCl3) for compound 6.

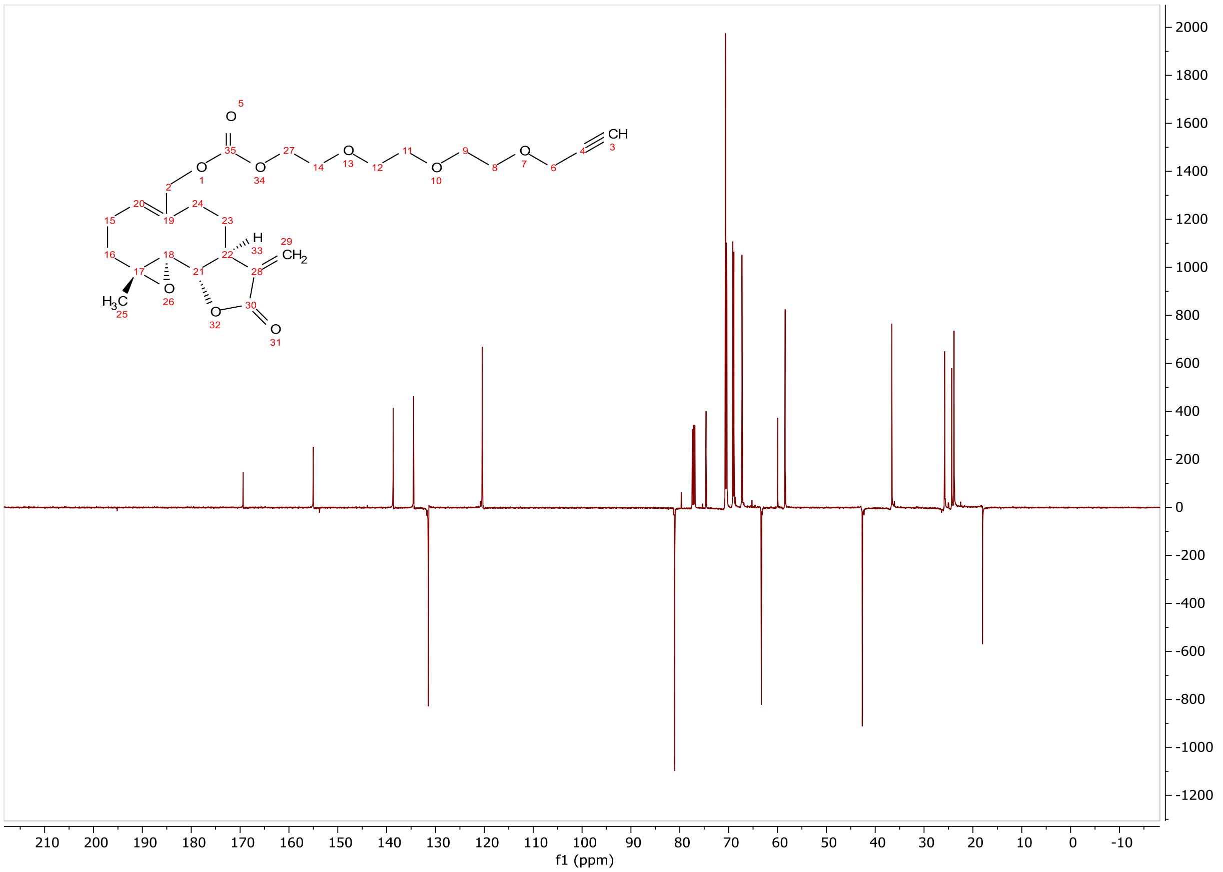

^{13}C NMR (126 MHz, CDCl3) for compound 6.

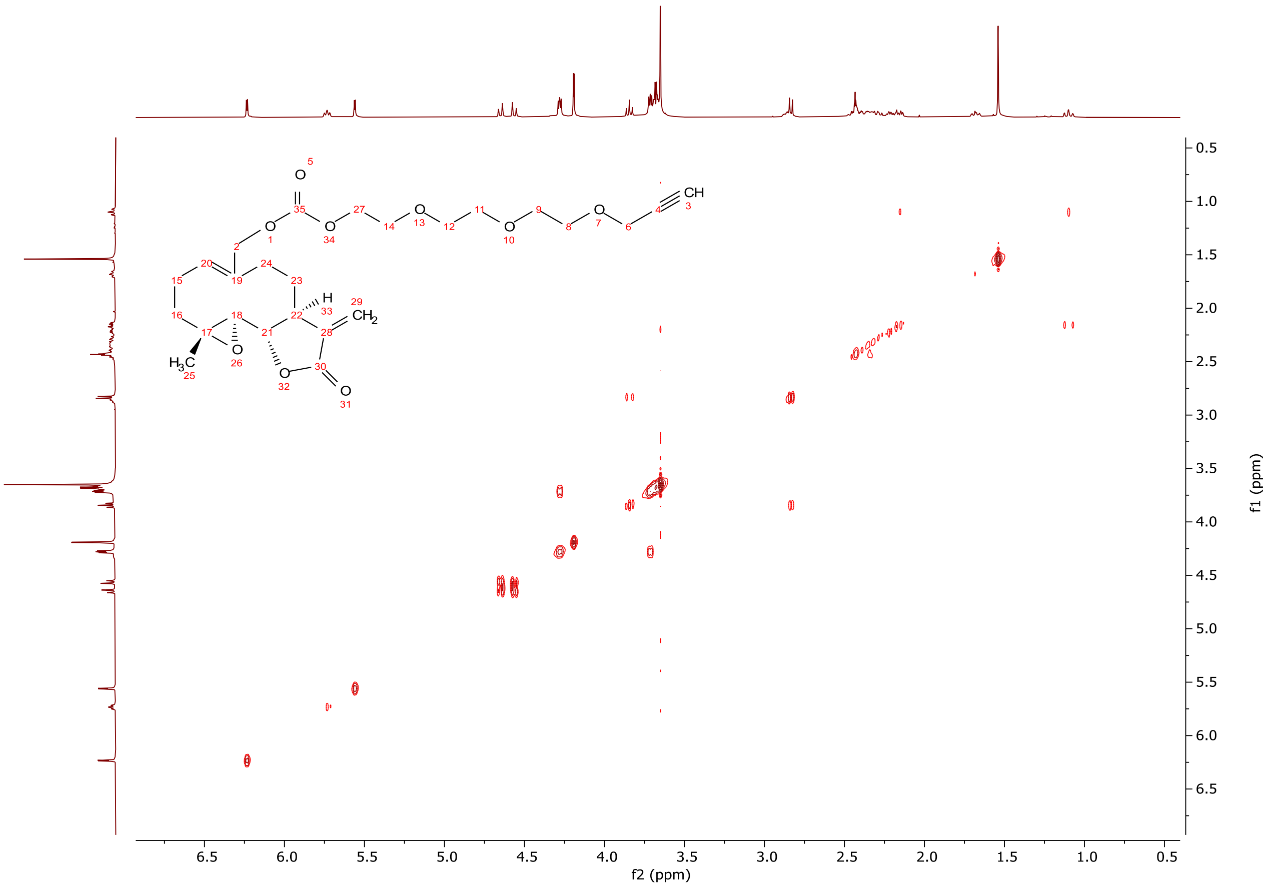

Cosy spectra for compound 6.

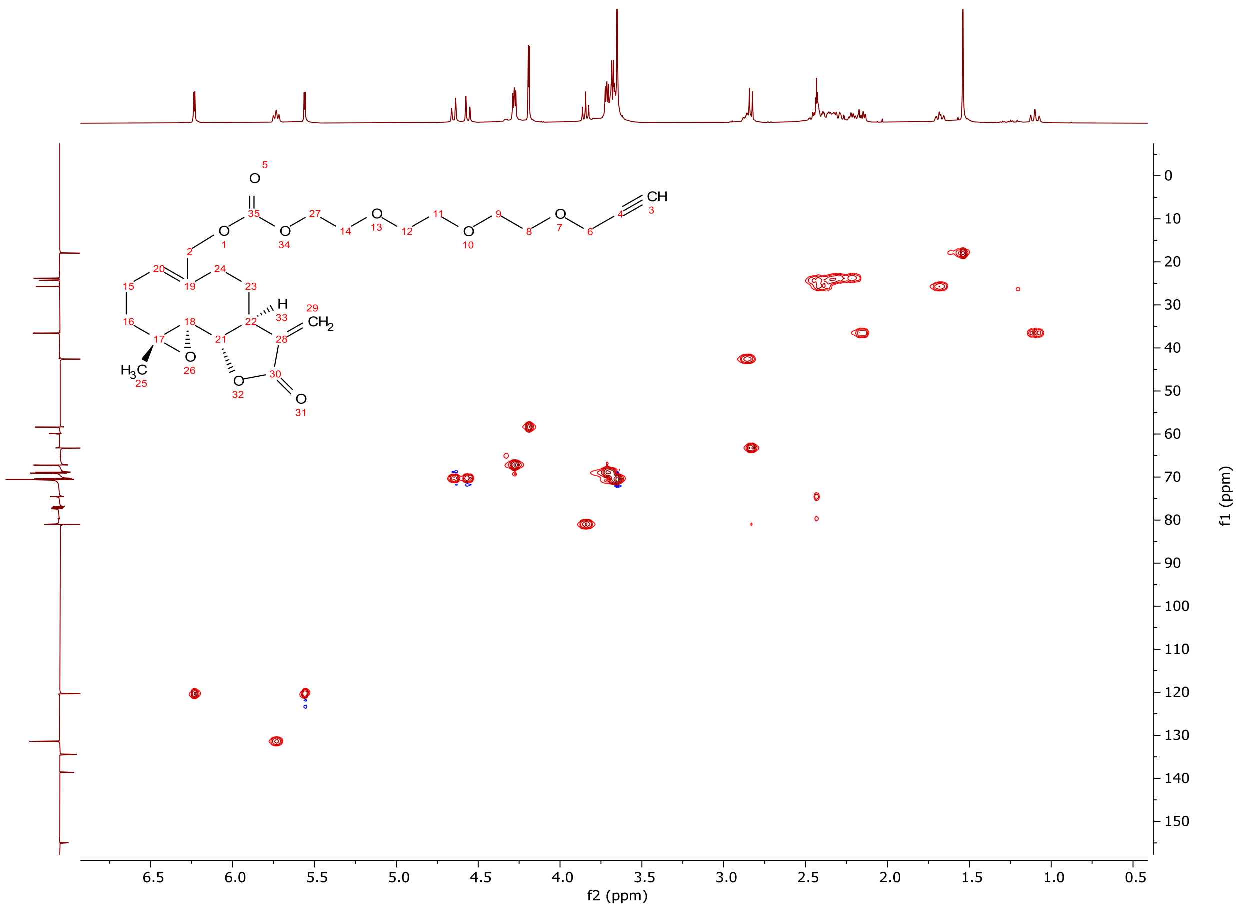

HSQC spectra for compound 6.
