# Supplementary material for: Parthenolide disrupts mitosis by inhibiting ZNF207/BUGZ-promoted kinetochore-microtubule attachment
Source: EMBO J. 2025 May 27;44(13):3764–93. doi: 10.1038/s44318-025-00469-2 (PMC12219771; doi:10.1038/s44318-025-00469-2)
Supplement: Supplementary file 2 — Table EV1 [file 44318_2025_469_MOESM2_ESM.docx]

**Table EV1.** List of proteins classified as Mitotic cell cycle process (GO: 1903047) and Mitotic cell cycle (GO: 0000278) detected in PTL pull-downs.

| **PROTEIN** | **FUNCTION IN MITOSIS** | **H/L RATIO** |
| --- | --- | --- |
| TK1 | Cell cycle regulated Thymidine kinase | 6 |
| NES | Regulates Intermediate Filaments during mitosis | 4,78 |
| CDK2 | Cell cycle kinase | 4,2 |
| EIF4EBP1 | Component of the protein complex eIF4F | 3,7 |
| PDCD6IP | ESCRT machinery. Required for completion of cytokinesis. | 3,46 |
| EIF4EBP1 | Component of the protein complex eIF4F | 3,7 |
| CHMP1A | Involved in cytokinesis. Involved in recruiting VPS4A and/or VPS4B to the midbody of dividing cells. | 3,34 |
| DIS3L2 | 3'-5'-exoribonuclease | 3,1 |
| ZNF207 | Recruits BUB3 and BUB1. Kinetochore-Microtubule attachment. | 3 |
| BCAT1 | Branched-chain-amino-acid aminotransferase. Required for Aurora B localization at centromeres. | 2,88 |
| TRIP13 | Mitotic Spindle Assembly Checkpoint signaling | 2,79 |
| KIF4A | Chromokinesin. | 2,7 |
| GSPT1 | GTPase component of the eRF1-eRF3-GTP ternary complex | 2,65 |
| MAP4 | Microtubule associated protein. | 2,64 |
| MSH2 | Component of the post-replicative DNA mismatch repair system | 2,56 |
| PPME1 | Demethylates PPP2CA. Controls spindle size. | 2,56 |
| TBCE | Tubulin chaperone. Enhances microtubule nucleation around chromatin in Drosophila. | 2,5 |
| ARL3 | Small GTP-binding protein. Required for normal cytokinesis. | 2,2 |
| CKAP5 | Microtubule and spindle assembly factor. | 2,1 |
| TPX2 | Spindle assembly factor | 2 |
