# Supplementary material for: Parthenolide disrupts mitosis by inhibiting ZNF207/BUGZ-promoted kinetochore-microtubule attachment
Source: EMBO J. 2025 May 27;44(13):3764–93. doi: 10.1038/s44318-025-00469-2 (PMC12219771; doi:10.1038/s44318-025-00469-2)
Supplement: Supplementary file 8 — Movie EV3 [file 44318_2025_469_MOESM8_ESM.zip › Movie EV3/Movie EV3.docx]

**Movie EV3:** Spinning-disk confocal live-cell imaging of dividing U2OS cells stably expressing H2B-GFP /mScarlet-α-tubulin undergoing indicated treatments. Time, hour:min.
