# Supplementary material for: Parthenolide disrupts mitosis by inhibiting ZNF207/BUGZ-promoted kinetochore-microtubule attachment
Source: EMBO J. 2025 May 27;44(13):3764–93. doi: 10.1038/s44318-025-00469-2 (PMC12219771; doi:10.1038/s44318-025-00469-2)
Supplement: Supplementary file 12 — Movie EV7 [file 44318_2025_469_MOESM12_ESM.zip › Movie EV7/Movie EV7.docx]

**Movie EV7:** Representative spinning disk confocal time-series of mitosis in HeLa cells expressing CKAP5-GFP, TPX2-GFP and AdV mCherry-KIF4A with AdV H2B-RFP or AdV H2B-GFP following indicated treatments. Time, hour:min.
