# Supplementary figures and images for: Parthenolide disrupts mitosis by inhibiting ZNF207/BUGZ-promoted kinetochore-microtubule attachment
Source: EMBO J. 2025 May 27;44(13):3764–93. doi: 10.1038/s44318-025-00469-2 (PMC12219771; doi:10.1038/s44318-025-00469-2)

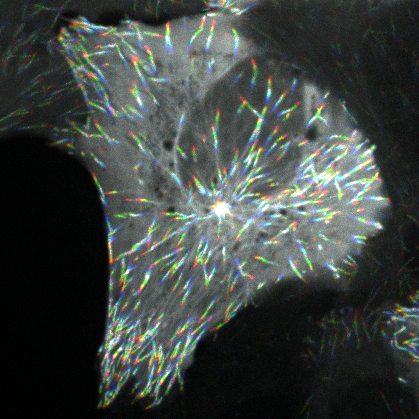

Supplement: Supplementary file 13 — Source data Fig. 1 [file 44318_2025_469_MOESM13_ESM.zip › SD figure 1/1B/DMSO.tif]

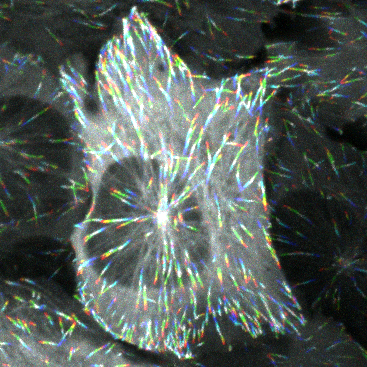

Supplement: Supplementary file 13 — Source data Fig. 1 [file 44318_2025_469_MOESM13_ESM.zip › SD figure 1/1B/PTL.tif]

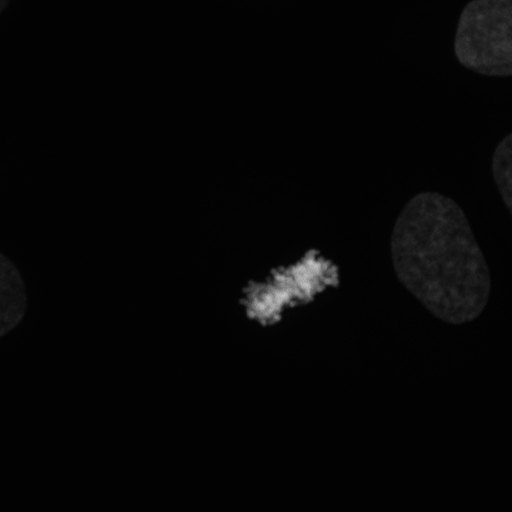

Supplement: Supplementary file 13 — Source data Fig. 1 [file 44318_2025_469_MOESM13_ESM.zip › SD figure 1/1G/Astral MT DMSO.tif]

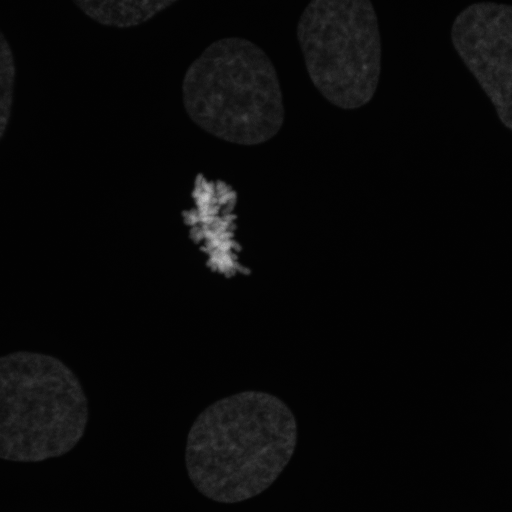

Supplement: Supplementary file 13 — Source data Fig. 1 [file 44318_2025_469_MOESM13_ESM.zip › SD figure 1/1G/Astral MT PTL.tif]

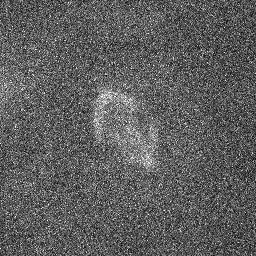

Supplement: Supplementary file 13 — Source data Fig. 1 [file 44318_2025_469_MOESM13_ESM.zip › SD figure 1/1H/MT TO DMSO.tif]

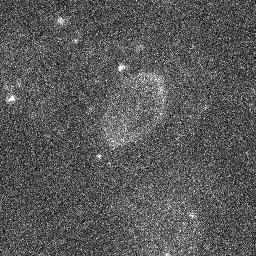

Supplement: Supplementary file 13 — Source data Fig. 1 [file 44318_2025_469_MOESM13_ESM.zip › SD figure 1/1H/MT TO PTL.tif]

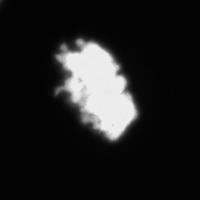

Supplement: Supplementary file 14 — Source data Fig. 2 [file 44318_2025_469_MOESM14_ESM.zip › SD figure 2/2C/DMSO.tif]

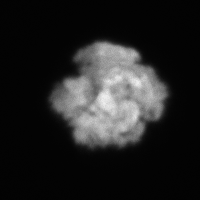

Supplement: Supplementary file 14 — Source data Fig. 2 [file 44318_2025_469_MOESM14_ESM.zip › SD figure 2/2C/PTL.tif]

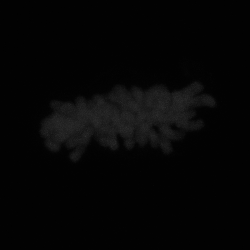

Supplement: Supplementary file 14 — Source data Fig. 2 [file 44318_2025_469_MOESM14_ESM.zip › SD figure 2/2E/DMSO.tif]

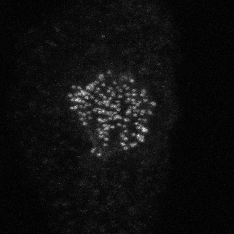

Supplement: Supplementary file 15 — Source data Fig. 3 [file 44318_2025_469_MOESM15_ESM.zip › SD figure 3/3A/Alkyne PTL 15uM.tif]

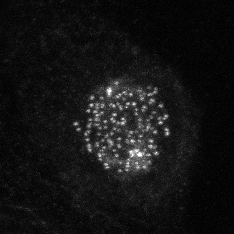

Supplement: Supplementary file 15 — Source data Fig. 3 [file 44318_2025_469_MOESM15_ESM.zip › SD figure 3/3A/Alkyne PTL 30uM.tif]

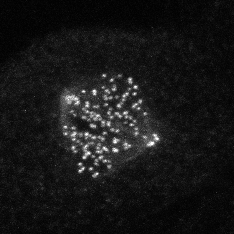

Supplement: Supplementary file 15 — Source data Fig. 3 [file 44318_2025_469_MOESM15_ESM.zip › SD figure 3/3A/Costunolide 15uM.tif]

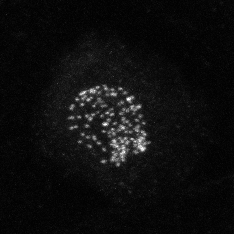

Supplement: Supplementary file 15 — Source data Fig. 3 [file 44318_2025_469_MOESM15_ESM.zip › SD figure 3/3A/Costunolide 30uM.tif]

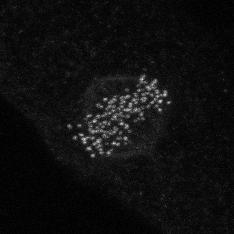

Supplement: Supplementary file 15 — Source data Fig. 3 [file 44318_2025_469_MOESM15_ESM.zip › SD figure 3/3A/DMSO.tif]

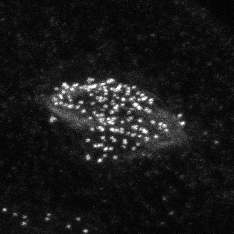

Supplement: Supplementary file 15 — Source data Fig. 3 [file 44318_2025_469_MOESM15_ESM.zip › SD figure 3/3A/PTL 15uM.tif]

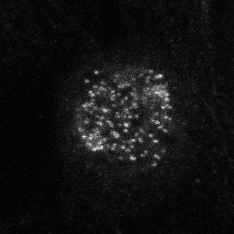

Supplement: Supplementary file 15 — Source data Fig. 3 [file 44318_2025_469_MOESM15_ESM.zip › SD figure 3/3A/PTL 30uM.tif]

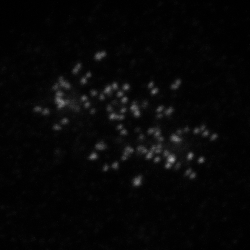

Supplement: Supplementary file 15 — Source data Fig. 3 [file 44318_2025_469_MOESM15_ESM.zip › SD figure 3/3C/Alkyne PTL at KT.tif]

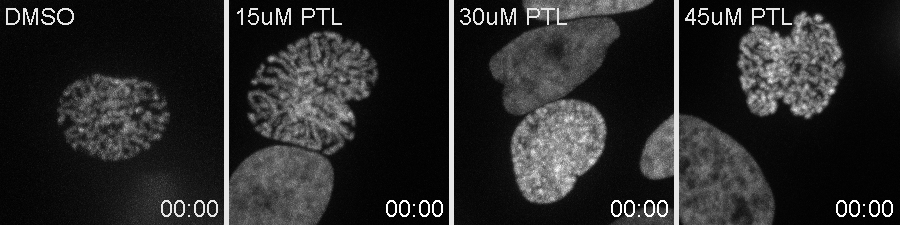

Supplement: Supplementary file 16 — Source data Fig. 4 [file 44318_2025_469_MOESM16_ESM.zip › SD figure 4/4B/HeLa GFP-BugZ_DMSO and PTL.tif]

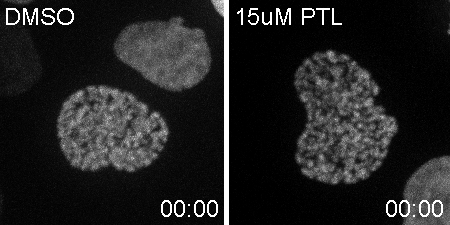

Supplement: Supplementary file 16 — Source data Fig. 4 [file 44318_2025_469_MOESM16_ESM.zip › SD figure 4/4B/HeLa Kyoto_DMSO and 15uM PTL.tif]

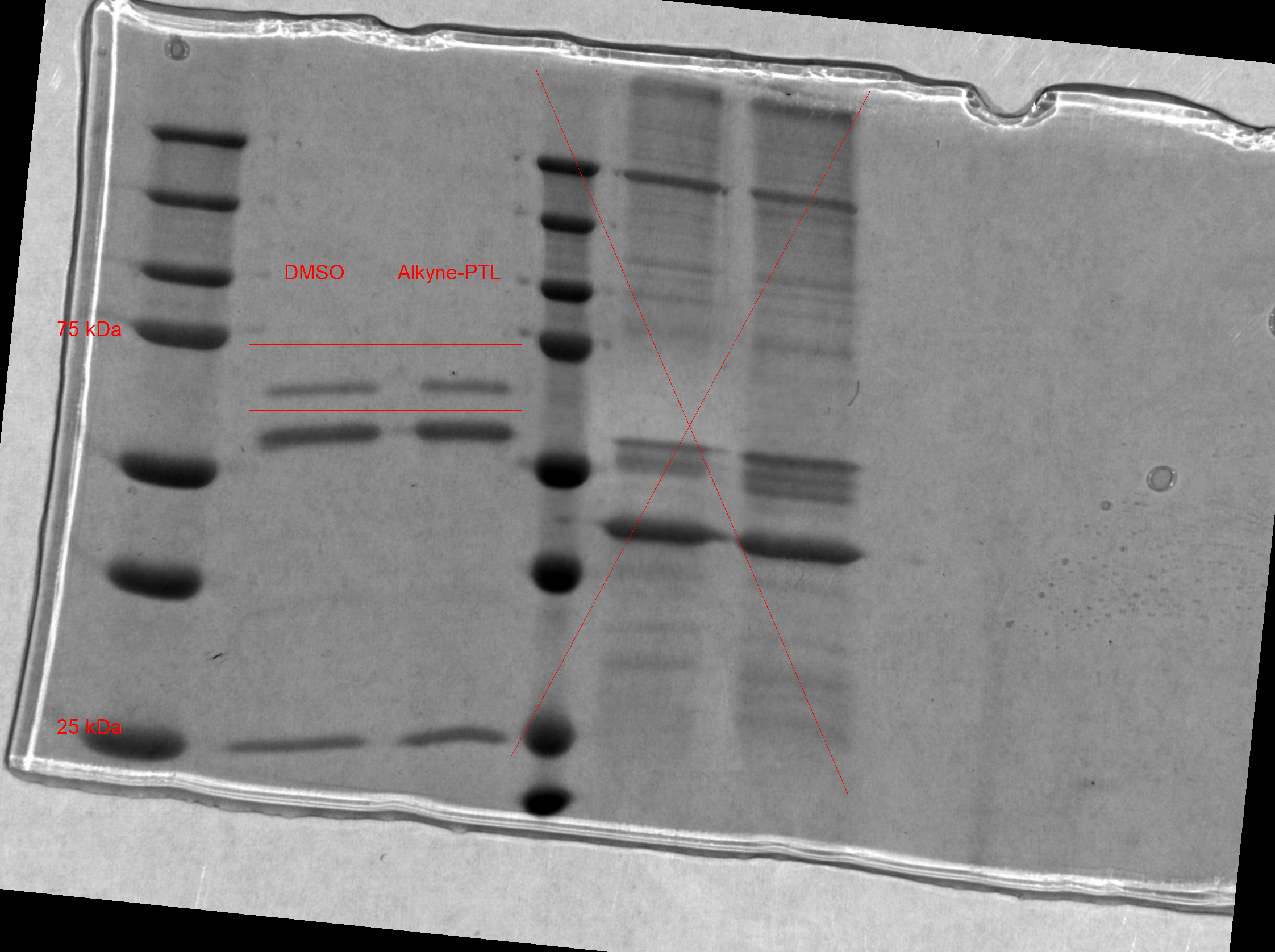

Supplement: Supplementary file 17 — Source data Fig. 5 [file 44318_2025_469_MOESM17_ESM.zip › SD figure 5/5A/Coomassie image.tif]

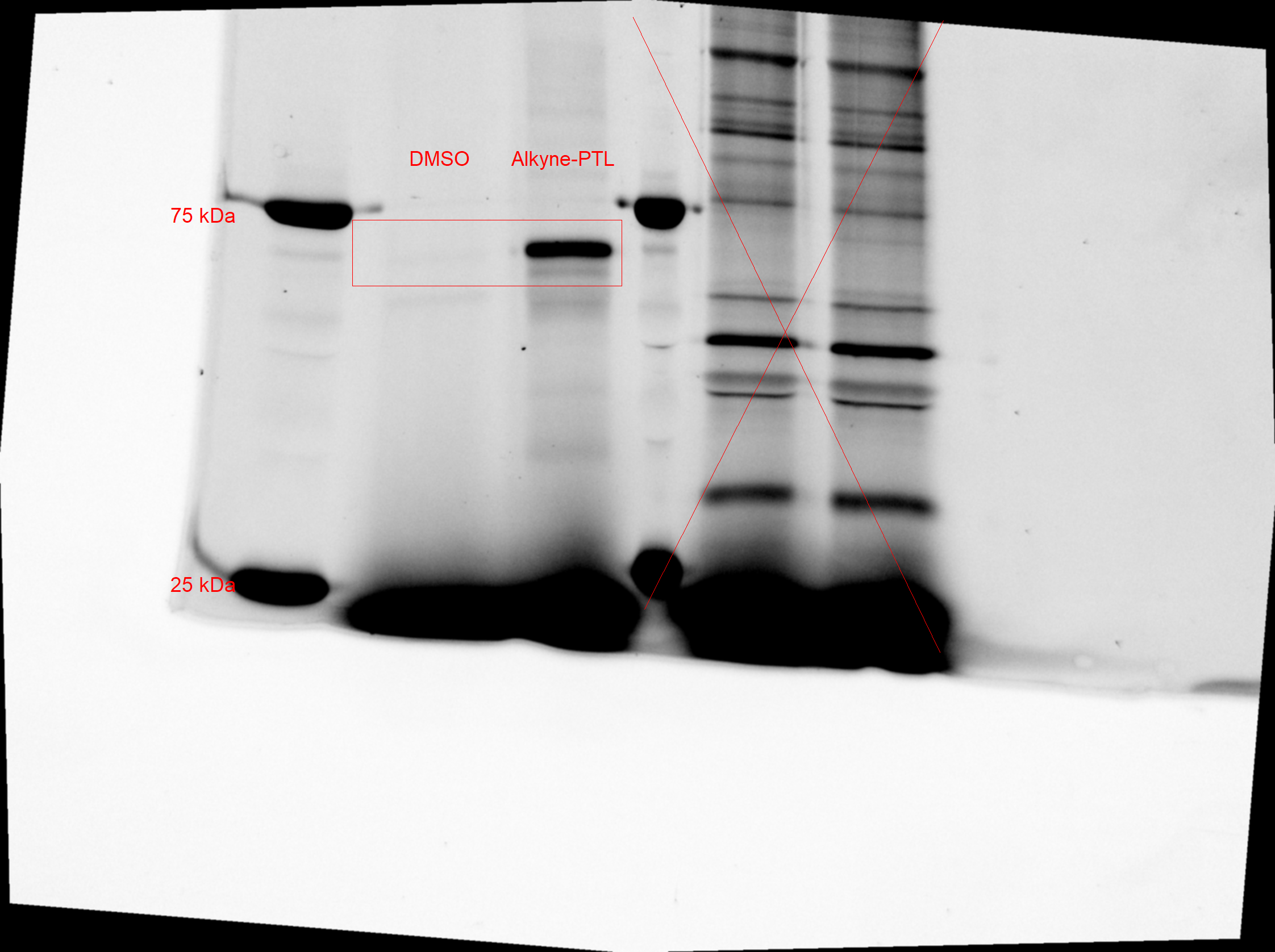

Supplement: Supplementary file 17 — Source data Fig. 5 [file 44318_2025_469_MOESM17_ESM.zip › SD figure 5/5A/Fluorescence image.tif]

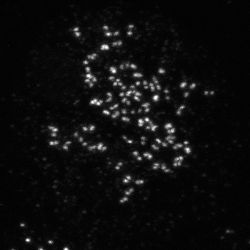

Supplement: Supplementary file 17 — Source data Fig. 5 [file 44318_2025_469_MOESM17_ESM.zip › SD figure 5/5B/siBUGZ.tif]

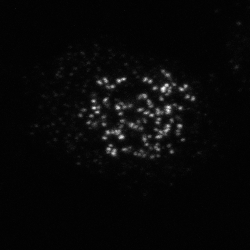

Supplement: Supplementary file 17 — Source data Fig. 5 [file 44318_2025_469_MOESM17_ESM.zip › SD figure 5/5B/siNT.tif]

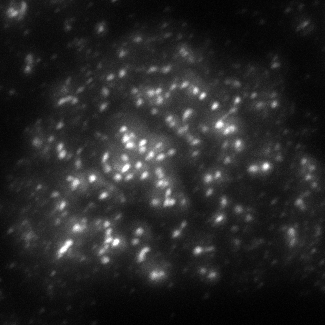

Supplement: Supplementary file 17 — Source data Fig. 5 [file 44318_2025_469_MOESM17_ESM.zip › SD figure 5/5D/DMSO.tif]

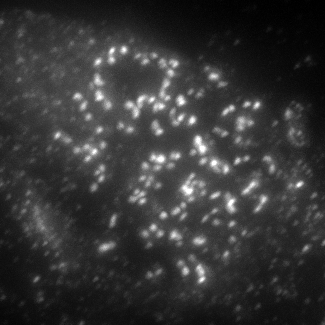

Supplement: Supplementary file 17 — Source data Fig. 5 [file 44318_2025_469_MOESM17_ESM.zip › SD figure 5/5D/PTL.tif]
