# Supplementary material for: Parthenolide disrupts mitosis by inhibiting ZNF207/BUGZ-promoted kinetochore-microtubule attachment
Source: EMBO J. 2025 May 27;44(13):3764–93. doi: 10.1038/s44318-025-00469-2 (PMC12219771; doi:10.1038/s44318-025-00469-2)
Supplement: Supplementary file 17 — Source data Fig. 5 [file 44318_2025_469_MOESM17_ESM.zip › SD figure 5/5F/Data deposited to the ProteomeXchange Consortium via the PRIDE partner repository.docx]

The mass spectrometry data used to identify modified BUGZ peptides have been deposited to the ProteomeXchange Consortium via the PRIDE partner repository with the dataset identifier PXD063178.
