# Supplementary material for: Parthenolide disrupts mitosis by inhibiting ZNF207/BUGZ-promoted kinetochore-microtubule attachment
Source: EMBO J. 2025 May 27;44(13):3764–93. doi: 10.1038/s44318-025-00469-2 (PMC12219771; doi:10.1038/s44318-025-00469-2)
Supplement: Supplementary file 18 — Expanded View Figures [file 44318_2025_469_MOESM18_ESM.pdf]

Expanded View Figures

Figure EV1. Alkyne-parthenolide and costunolide phenocopy the mitotic effects of parthenolide.

(**A**) Molecular structures of PTL, alkyne-PTL derivative and costunolide. (**B**) Representative confocal maximum projections of cold stable microtubules from U2OS cells treated with the indicated compounds (15 μM) and subjected to a short cold treatment. Scale bar: 10 μm. (**C**) Quantification of α-tubulin intensity at the mitotic spindle of cells treated as indicated in (**B**). *N*, *n* (*N* = number of cells, *n* = number of experiments): DMSO (57, 3), 15 μM alkyne-PTL (58, 3), 15 μM costunolide (39, 3). ****$p \leq 0.0001$. (**D**) Representative confocal max projections of mitotic U2OS cells undergoing the indicated treatments. Scale bar: 10 μm. (**E**) Quantification of astrin intensity at aligned kinetochores normalized to CENP-C intensity for DMSO, 15 μM alkyne-PTL and 15 μM costunolide treated cells. *N*, *n* (*N* = number of cells, *n* = number of experiments): DMSO (30, 3), 15 μM alkyne-PTL (30, 3), 15 μM costunolide (30, 3). ****$p \leq 0.0001$. (**F**) Immunoblot of α-tubulin detyrosination levels in U2OS cells undergoing the stated treatments. Quantification of the relative levels of detyrosination in cells undergoing the stated treatments. ****$p \leq 0.0001$, **$p \leq 0.01$. Replicates are color-coded for all quantifications. All data are presented as mean ± SD values from three independent replicates. Statistical analysis was performed using unpaired t-test with Welch's correction.

© The Author(s)

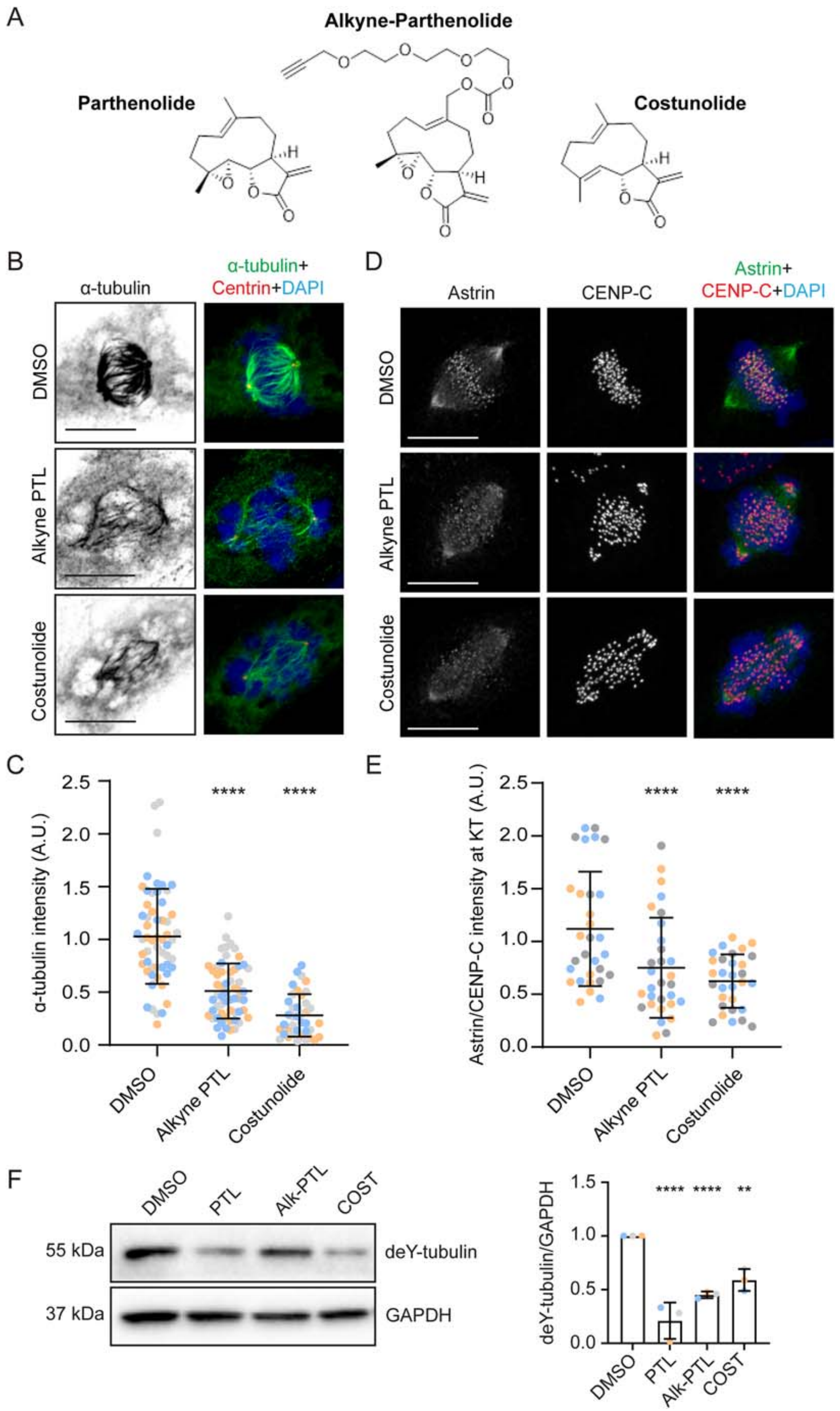
A
Alkyne-Parthenolide
Parthenolide
Costunolide
B
α-tubulin
α-tubulin+Centrin+DAPI
DMSO
Alkyne PTL
Costunolide
D
Astrin
CENP-C
Astrin+CENP-C+DAPI
C
α-tubulin intensity (A.U.)
****
E
Astrin/CENP-C intensity at KT (A.U.)
F
DMSO PTL Alk-PTL COST
55 kDa
deY-tubulin
37 kDa
GAPDH
deY-tubulin/GAPDH
**** **** **

 © The Author(s)

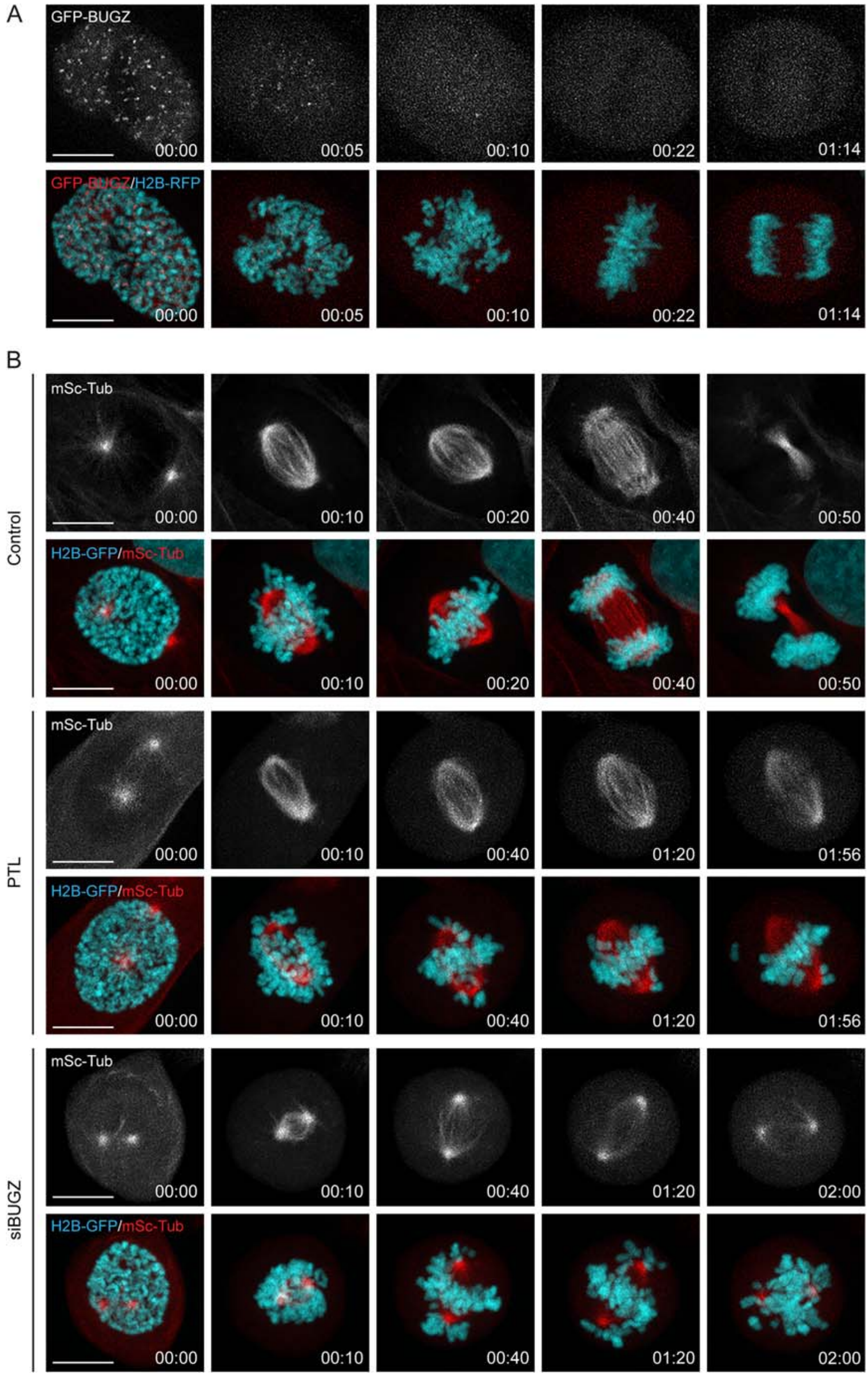
A
GFP-BUGZ
00:00
00:05
00:10
00:22
01:14
GFP-BUGZ/H2B-RFP
00:00
00:05
00:10
00:22
01:14
B
mSc-Tub
00:00
00:10
00:20
00:40
00:50
Control
H2B-GFP/mSc-Tub
00:00
00:10
00:20
00:40
00:50
mSc-Tub
00:00
00:10
00:40
01:20
01:56
PTL
H2B-GFP/mSc-Tub
00:00
00:10
00:40
01:20
01:56
mSc-Tub
00:00
00:10
00:40
01:20
02:00
siBUGZ
H2B-GFP/mSc-Tub
00:00
00:10
00:40
01:20
02:00

Figure EV2. BUGZ localizes at kinetochores in early mitosis and its depletion induces mitotic defects similar to parthenolide treatment.

(**A**) Representative spinning-disk confocal time-series of mitosis in HeLa cells stably expressing GFP-BUGZ and infected with adenovirus to express H2B-RFP. Scale bar: 10 µm. (**B**) Representative spinning-disk confocal time-series of mitosis in control, 15 µM PTL- and siBUGZ-treated U2OS cells stably expressing H2B-GFP/mScarlet-α-tubulin. Scale bar: 10 µm.

 © The Author(s)

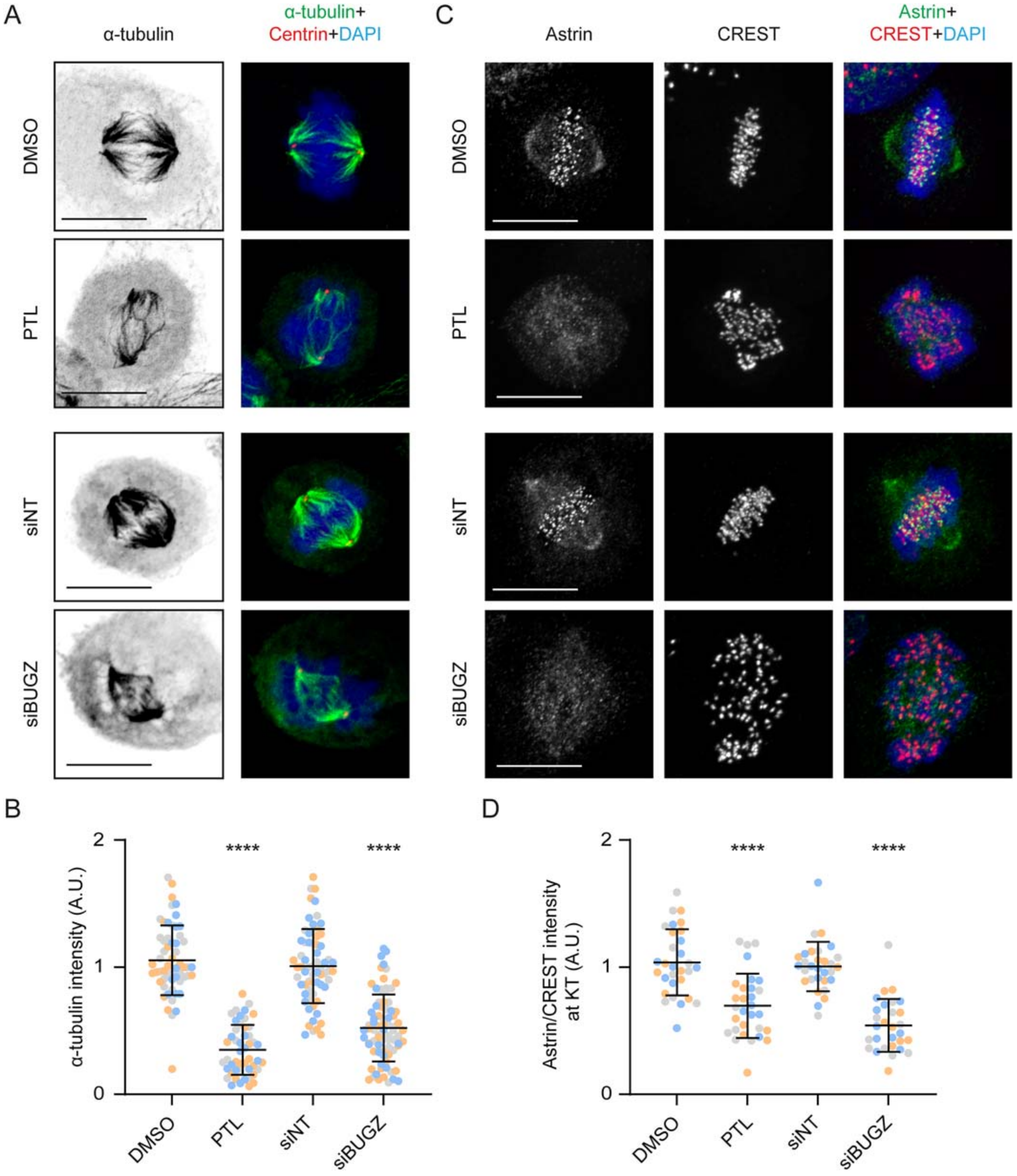
A
α-tubulin
α-tubulin+
Centrin+DAPI
DMSO
PTL
siNT
siBUGZ
C
Astrin
CREST
Astrin+
CREST+DAPI
DMSO
PTL
siNT
siBUGZ
B
α-tubulin intensity (A.U.)
2
1
0
****
****
DMSO
PTL
siNT
siBUGZ
D
Astrin/CREST intensity at KT (A.U.)
2
1
0
****
****
DMSO
PTL
siNT
siBUGZ

Figure EV3. BUGZ depletion disrupts kinetochore-microtubule attachments in a manner similar to parthenolide treatment.

(**A**) Representative confocal max projections of cold stable microtubules from HeLa cells under the indicated conditions. Scale bar: 10 μm. (**B**) Quantification of α-tubulin intensity at the mitotic spindle of the cells treated as indicated in (**A**). *N*, *n* (*N* = number of cells, *n* = number of experiments): DMSO (57, 3), 15 μM PTL (49, 3), siNT (66, 3), siBUGZ (79, 3). ****$p \le 0.0001$. (**C**) Representative confocal max projections of mitotic cells subjected to the indicated treatments. Scale bar: 10 μm. (**D**) Quantification of astrin intensity at aligned kinetochores normalized to CREST intensity for the conditions indicated in (**C**). *N*, *n* (*N* = number of cells, *n* = number of experiments): DMSO (30, 3), 15 μM PTL (30, 3), siNT (30, 3), siBUGZ (29, 3). ****$p \le 0.0001$. Replicates are color coded for all quantifications. All data are presented as mean ± SD values from three independent replicates. Statistical analysis was performed using unpaired t-test with Welch's correction and unpaired t-test for PTL and siBUGZ, respectively, in (**B**). Unpaired t-test and Mann-Whitney test were used for PTL and siBUGZ, respectively, in (**D**).

 © The Author(s)

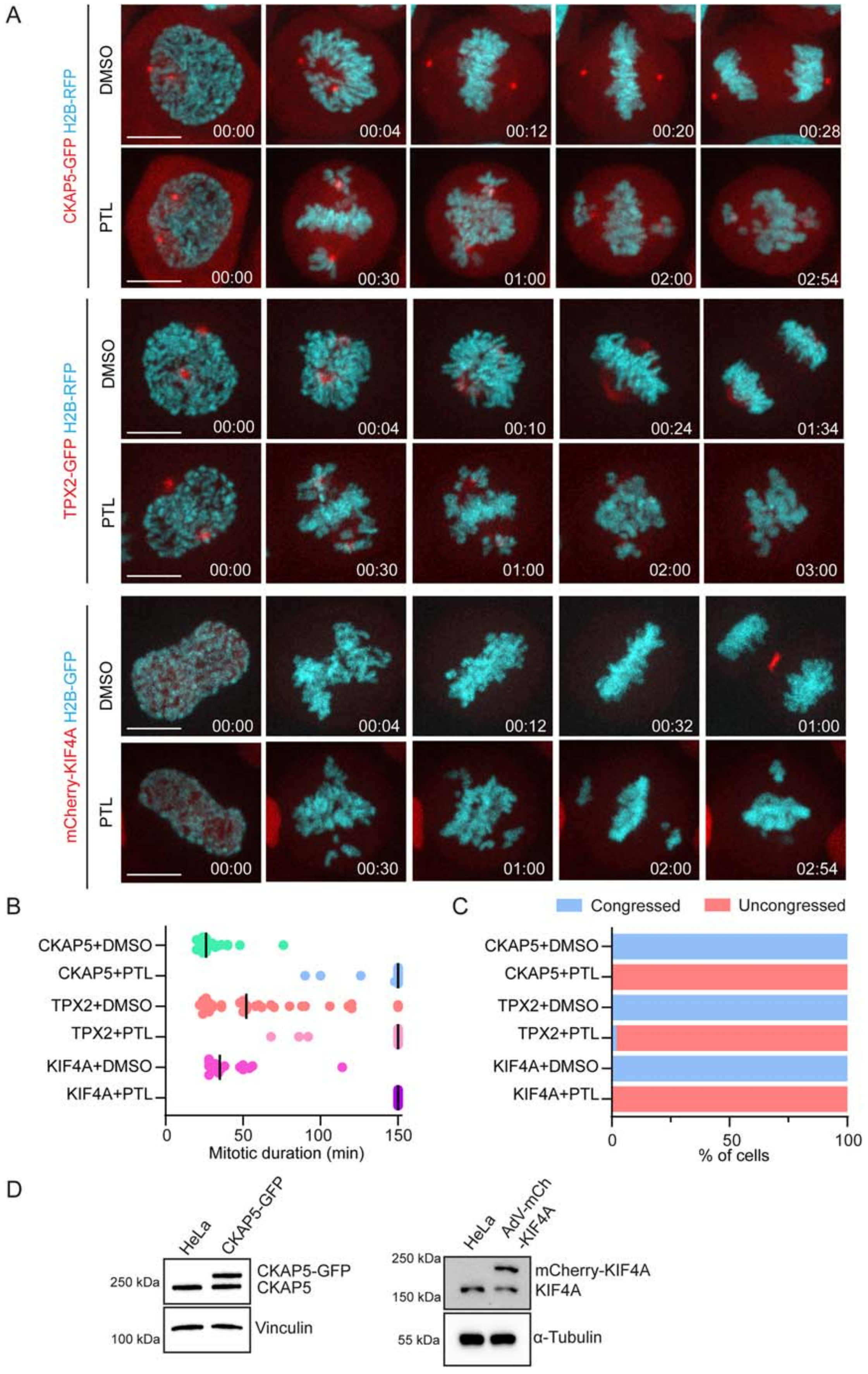
A
CKAP5-GFP H2B-RFP
DMSO
PTL
00:00
00:04
00:12
00:20
00:28
00:00
00:30
01:00
02:00
02:54
TPX2-GFP H2B-RFP
DMSO
PTL
00:00
00:04
00:10
00:24
01:34
00:00
00:30
01:00
02:00
03:00
mCherry-KIF4A H2B-GFP
DMSO
PTL
00:00
00:04
00:12
00:32
01:00
00:00
00:30
01:00
02:00
02:54
B
CKAP5+DMSO
CKAP5+PTL
TPX2+DMSO
TPX2+PTL
KIF4A+DMSO
KIF4A+PTL
0
50
100
150
Mitotic duration (min)
C
Congressed
Uncongressed
CKAP5+DMSO
CKAP5+PTL
TPX2+DMSO
TPX2+PTL
KIF4A+DMSO
KIF4A+PTL
0
50
100
% of cells
D
HeLa
CKAP5-GFP
250 kDa
100 kDa
CKAP5-GFP
CKAP5
Vinculin
HeLa
AdV-mCh -KIF4A
250 kDa
150 kDa
55 kDa
mCherry-KIF4A
KIF4A
α-Tubulin

Figure EV4. Overexpression of CKAP5, TPX2 or KIF4A cannot reverse the mitotic defects induced by parthenolide.

(**A**) Representative spinning-disk confocal time-series of mitosis in DMSO- and 15 μM PTL-treated HeLa cells stably expressing CKAP5-GFP, TPX2-GFP and HeLa cells transduced for expression of mCherry-KIF4A undergoing infection with adenovirus to express H2B-RFP or H2B-GFP. Scale bar: 10 μm. (**B**) Quantification of mitotic duration and chromosome congression status (**C**) in cells with indicated conditions in (**A**). Median is plotted for mitotic duration. *N*, *n* (*N* = number of cells, *n* = number of experiments) for congression phenotype: CKAP5-GFP + DMSO (50, 3), CKAP5-GFP + 15 μM PTL (45, 3), TPX2-GFP + DMSO (50, 3), TPX2-GFP + 15 μM PTL (45, 3), mCherry-KIF4A + DMSO (26, 3), mCherry-KIF4A + 15 μM PTL (22, 3); for mitotic duration: CKAP5-GFP + DMSO (32, 3), CKAP5-GFP + 15 μM PTL (37, 3), TPX2-GFP + DMSO (32, 3), TPX2-GFP + 15 μM PTL (16, 3), mCherry-KIF4A + DMSO (16, 3), mCherry-KIF4A + 15 μM PTL (17, 3). (**D**) Immunoblots for cellular expression levels of CKAP5-GFP and mCherry-KIF4A.

 © The Author(s)

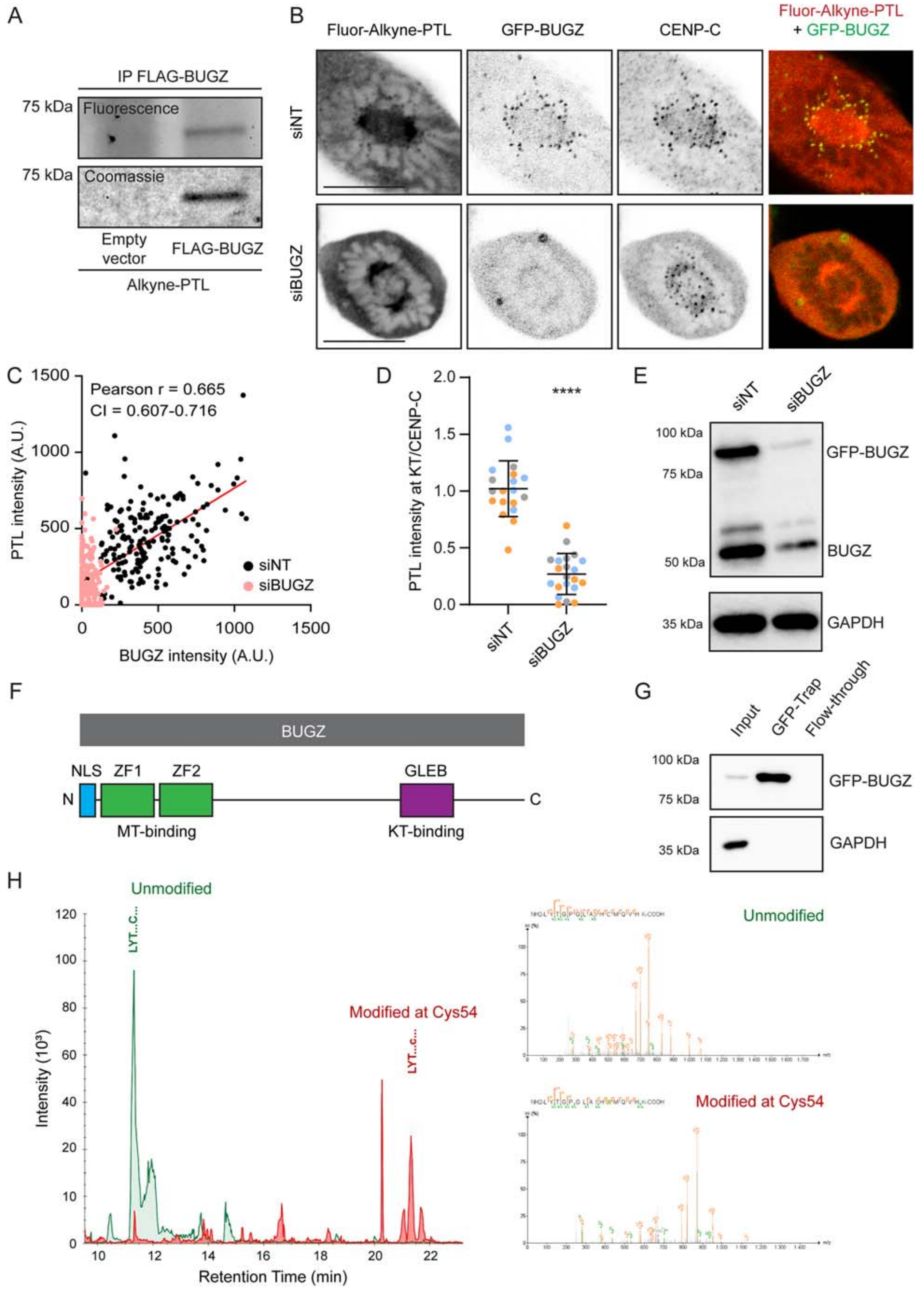
A
IP FLAG-BUGZ
75 kDa Fluorescence
75 kDa Coomassie
Empty vector
FLAG-BUGZ
Alkyne-PTL
B
Fluor-Alkyne-PTL
GFP-BUGZ
CENP-C
Fluor-Alkyne-PTL + GFP-BUGZ
siNT
siBUGZ
C
Pearson r = 0.665
CI = 0.607-0.716
PTL intensity (A.U.)
BUGZ intensity (A.U.)
siNT
siBUGZ
D
PTL intensity at KT/CENP-C
****
siNT
siBUGZ
E
siNT
siBUGZ
100 kDa
75 kDa
50 kDa
35 kDa
GFP-BUGZ
BUGZ
GAPDH
F
BUGZ
NLS
ZF1
ZF2
GLEB
N
C
MT-binding
KT-binding
G
Input
GFP-Trap
Flow-through
100 kDa
75 kDa
35 kDa
GFP-BUGZ
GAPDH
H
Unmodified
LYT...C...
Modified at Cys54
LYT...c...
Intensity (10³)
Retention Time (min)
Unmodified
Modified at Cys54

© The Author(s)

Figure EV5. Parthenolide localizes at kinetochores by covalently binding Cys54 of BUGZ.

(**A**) Fluorencence and Comassie staining of immunoprecipitated FLAG empty and FLAG-BUGZ from HEK 293T cells treated alkyne-PTL. (**B**) Representative confocal images of click-based imaging of 5 µM fluor-Cy5-alkyne-PTL in HeLa GFP-BUGZ cells under the indicated conditions. Scale bar: 10 µm. (**C**) Scatter plot showing the intensity of BUGZ (x-axis) and 5 µM fluor-Cy5-alkyne-PTL (y-axis) at individual kinetochores from the indicated conditions in (**B**). Each dot represents a single kinetochore. A Pearson correlation line is shown for the correlation between BUGZ and fluor-Cy5-alkyne-PTL levels. (**D**) Quantification of 5 µM fluor-Cy5-alkyne-PTL intensity at kinetochores normalized to CENP-C intensity for the conditions indicated in (**B**). *N*, *n* (number of cells, number of experiments): siNT (19, 3) siBUGZ (21, 3). ****$p \leq 0.0001$. Replicates are color coded. Data are presented as mean ± SD values from three independent replicates. Statistical analysis was performed using unpaired t-test. (**E**) Immunoblot for BUGZ depletion efficiency in HeLa GFP-BUGZ cells. (**F**) Illustration of domain architecture of BUGZ. (**G**) Western-blot with anti-BUGZ antibody of in cellulo GFP-Trap pulldown sample from HeLa cells stably expressing GFP-BUGZ treated with 50 µM PTL. (**H**) Extracted ion chromatograms and MS-MS spectra for peptides with and without PTL modification at Cys54 (red and green, respectively) from the GFP-Trap sample shown in (**G**).

 © The Author(s)
